# Revealing the Microbiome of Four Different Thermal Springs in Turkey with Environmental DNA Metabarcoding

**DOI:** 10.3390/biology11070998

**Published:** 2022-06-30

**Authors:** Işılay Çelik, Emre Keskin

**Affiliations:** 1 Biotechnology Institute, Ankara University, Ankara 06135, Turkey; isilaycelik@ankara.edu.tr; 2 Evolutionary Genetics Laboratory (eGL), Department of Fisheries and Aquaculture, Agricultural Faculty, Ankara University, Ankara 06135, Turkey

**Keywords:** microbiome, environmental DNA (eDNA), metabarcoding, thermal spring, high throughput sequencing, extremophiles

## Abstract

**Simple Summary:**

The physicochemical conditions of thermal springs are one of the most significant barriers for detecting microbial life. According to the findings of various studies, high-throughput DNA sequencing technology can be utilized to perform more precise and detailed microbiome assessments. The main goal of this paper was to determine the microbiome in a thermal spring by metabarcoding environmental DNA obtained from four different sources and revealing how temperature and chemical composition affect the microbiome. This research also aimed to gather information that will aid in determining the best gene region and bioinformatic pipeline. The findings revealed a link between four different thermal springs’ physicochemical parameters and microbial composition and we found various manipulable steps in this study. This research is also first comprehensive thermal spring metabarcoding study conducted in Turkey.

**Abstract:**

One of the most significant challenges for detecting microbial life in thermal springs by conventional techniques such as culturing is these places’ physicochemical (temperature, heavy metal content, pH, etc.) conditions. Data from several studies suggest that high-throughput DNA sequencing technologies can be used to perform more accurate and detailed microbiome analyses. The primary aim of this paper was to determine the microbiome in the thermal source by metabarcoding environmental DNA isolated from four different sources and reveal the reflection of differences caused by temperature and chemical content on the microbiome. DNA was extracted from water filtered with enclosed filters and using the Illumina high-throughput sequencing platform, V3 and V4 regions of the 16S rRNA gene were sequenced. The results showed a correlation between physicochemical conditions and microorganism composition of four different thermal springs. Springs with extremely high temperature (89–90 °C) were dominated by hyperthermophiles such as *Hydrogenobacter* and *Thermus*, while a spring with a high temperature (52 °C) was dominated by thermophiles such as *Thermoanaerobaculum* and *Desulfurispora*, and a spring with a low temperature (26 °C) and high salinity was dominated by halophiles and sulfur-oxidizers such as *Hydrogenovibrio* and *Sulfirimonas*. With this research, we observed many manipulable steps according to the work of interest. This study sought to obtain data that will help decide the right gene region and choose the optimal bioinformatic pipeline.

## 1. Introduction

The entire genetic material of microorganisms in a particular niche is defined as the microbiome [1]. Microbial communities represent the world’s earliest inhabitants and shape the dynamics of environments ranging from the mammalian digestive tract to hydrothermal vents [2]. It covers a broad spectrum from nutrient cycling to the protection of human health [3,4]. For this reason, the detection, classification, and analysis of these communities that live and work together in a particular environment gain importance in a wide range of studies, from clinical studies to biotechnological applications [5,6]. The methods used in microbiome research are generally divided into two: conventional and molecular techniques [7]. Studies using culture, microscope, and serological analysis constitute conventional techniques. Molecular phylogenetic studies have shown that less than 1% of bacterial diversity in any environmental sample can be cultured in the laboratory [8]. This loss of diversity, which will be experienced with the culture method, will cause problems in the analysis of microbial populations [9]. In addition to conventional methods, molecular techniques such as 16S ribosomal RNA analysis are also used [10]. Modern microbiome studies generally rely on the analysis of 16S rRNA sequences for taxonomic identification of bacterial and archaeal strains. Such analyses have become the standard for prokaryotic taxonomy [11]. Microbiome analysis is performed more accurately and in detail thanks to the developments in high-throughput DNA sequencing and data analysis technologies used in recent years with new methods [12]. The amplification technique of the 16S rRNA gene combined with high-throughput DNA sequencing allows for deep investigations of microbial communities [13]. Molecular techniques in microbial diversity studies to be carried out in environmental samples yield more comprehensive results compared with conventional techniques [14]. Direct sequence analysis of DNA obtained from a particular environmental sample is one of the most valuable methods for assessing the microbial community structure of that sample [15]. It does not involve selection or enrichment as conventional techniques and minimizes technical errors.

Environmental DNA (eDNA) refers to DNA obtained from organic wastes such as mucus, skin, feces, or biofilm, isolated from environmental samples (such as soil, air, or water) without the direct need of any living organism [16]. eDNA is a fast, economical, and effective method for multi-species detection and searching for species richness. Environmental DNA (eDNA) offers a unique opportunity to isolate and identify genetic material from the environment and assess biodiversity at increased speed and lower cost than conventional methods [17]. With these advantages, the eDNA method has become one of the best methods for microbiome analysis. The barcoding method is frequently used to determine species from the obtained eDNA samples [18]. For DNA replication and sequencing, certain regions on the nuclear and organelle DNAs were defined and standardized under ‘DNA barcoding’. The most widely used and standardized DNA barcode regions in the literature are the mitochondrial cytochrome c oxidase I (COI) gene for animals, ribulose 1,5-biphosphate 4 carboxylase (rbcL), and maturase K (matK) genes for plants, the ITS gene for fungi, and 16S ribosomal RNA (16S rRNA) gene for microorganisms [19]. The 16S rRNA gene, frequently used in microbiome analysis, is approximately 1500 base pairs long. This gene contains nine hypervariable regions that often vary between bacterial and archaeal species and can be used in species identification [20]. The 16S rRNA gene, a highly conserved region, is widely used in environmental microbiology, molecular evolution, microorganism classification, and phylogenetic analysis. With the eDNA barcoding method, the relevant barcode region of a single species from the environmental DNA sample is amplified by polymerase chain reaction through primers specially designed for the living group, and species detection is performed using methods such as qPCR and Sanger sequencing. Detection of multiple species is carried out through universal primers targeting shorter regions, and this method is called ‘eDNA metabarcoding’. Amplified DNA regions by the polymerase chain reaction established with metabarcoding primers, shorter and more numerous than the barcoding method, are analyzed using high-throughput sequencing technologies [17]. High-throughput sequencing technologies are becoming the most suitable method in microbiome metabarcoding studies using 16S rRNA, as they can make millions of reads simultaneously, their output can be detected directly, and they are less costly and faster than the first generation.

Turkey is located on the tectonically active Alpine–Himalayan Belt. This tectonic activity, which brings along a high number of young fault lines and volcanic formations, also causes the formation of geothermal potential in Turkey. Underground spring waters with a temperature above 20 °C are called thermal springs. There are approximately 1000 thermal springs of natural origin, with different characteristics (flow rate, radioactivity, molten mineral ratios, and accessibility) and temperatures throughout Turkey. Turkey is the first in Europe in terms of thermal springs due to its number and qualities and is among the top five countries in the world. It has been reported that thermal springs support a rich microbial diversity and constitute natural niches for microorganisms such as thermophilic (>50 °C) and hyperthermophilic (>80 °C) ecosystems [21]. Due to the technical problems in providing ideal culture conditions, these groundwaters, which are the habitat of many microorganisms, still contain biodiversity that has not been fully classified. Defining this biodiversity is very important both for detecting possible new microorganism species and for the use of the specific functions of the detected microorganisms (fermentation, energy production, biological treatment) and the compounds they produce (bioactive compounds, acid, enzyme, pigment) in biotechnological research. Considering that the oldest life forms on Earth are organisms adapted to living in extreme conditions, thermal springs with extreme conditions can provide information about the origin and early evolution of life. It also helps define the limits on which life can be sustained [9]. For these reasons, the determination of the thermal source microbiome is of great importance.

In this study, the goal of detecting and identifying the microbiome was achieved using eDNA metabarcoding and next-generation sequencing methods. Water samples from four thermal springs were filtered and extracted. Hypervariable regions of 16S rRNA (V3 and V4) were amplified, and amplicons were sequenced with Illumina platform. “fastq” files were processed with modified ObiTools pipeline and SILVAngs [22,23]. Comparisons according to both 16S rRNA regions and different bioinformatic workflow modifications were carried out. It is observed that each variable in this research has a different effect on the specification of the microbiome. In addition, with this study, microbiomes of thermal springs in Turkey are revealed for the first time.

## 2. Materials and Methods

### 2.1. Site Description and Sample Collection

The thermal springs located in Nevşehir-Kozaklı (39°12′43.2″ N, 34°51′46.8″ E), Ankara-Kızılcahamam (40°28′30″ N, 32°39′16″ E), Yozgat-Boğazlıyan (39°11′42″ N, 35°11′22″ E), and Muğla-Dalaman (36°41′44″ N, 28°47′31″ E) were chosen for this research. Water samples were collected in sterile 2-liter bottles in triplicates and brought to the laboratory for further analysis immediately. To detect cross-contamination, negative samples for each thermal spring were prepared with UV-treated ultrapure water. Using tubings and a peristaltic pump, each sample was filtered through 0.22 μM Sterivex enclosed filters. After filtration, the inlet and outlet of the Sterivex filters were sealed and stored at −30 °C until further processing.

### 2.2. Physicochemical Analysis of Water Samples

The physicochemical analysis reports were taken from official institution reports of Mineral Research and Exploration (MTA) and Republic of Turkey Ministry of Health. According to the results of physicochemical analysis, Nevşehir-Kozaklı and Ankara-Kızılcahamam thermal springs have very hot, Yozgat-Boğazlıyan has hot, and Muğla-Dalaman has low temperature; in addition to the temperature factor, it has been observed that these sources create an extreme environment in terms of parameters such as bicarbonate, sulfate, and nitrate (Supplementary Table S1).

### 2.3. DNA Extraction and PCR Amplifications

According to the manufacturer’s instructions, DNA was extracted directly from Sterivex filters using the Qiagen Blood & Tissue DNA Purification Kit with slight modifications (Supplementary Data S1). The DNA quantity and quality of the obtained DNA isolates were measured with the Colibri Microvolume Spectrometer device.

The 16S rDNA genes of hypervariable regions 16S V3 and V4 were amplified using the following specific primers: 16sV3F [24] (ACTCCTACGGGAGGCAGCAGT) and 16sV3R [25] (ACCGCGGCTGCTGGCAC), 515F-Y [26] (GTGYCAGCMGCCGCGGTAA) and 806R-B [27] (GGACTACNVGGGTWTCTAAT). PCR was performed with a primary heating step for 2 min at 94 °C, followed by 30 cycles of denaturation for 1 min at 94 °C, annealing for 45 s at 53 °C, and extension for 1 min at 72 °C, then followed by a final extension step for 5 min at 72 °C. Each 10 μL reaction mixture contained 2 μL of 5× Promega Colorless GoTaq Flexi buffer, 1:10 and 1:100 diluted 0.5 μL DNA, 1 μL of 25 mM MgCl_2_, 0.8 μL of 3.2 μM dNTP, 0.5 μL of each primer (5 pmol/mL), 0.1 μL of Taq DNA polymerase, and sterile water to a final volume of 10 μL. For each field and primer, DNA concentration was optimized with serial dilutions as 1:10 and 1:100. For the dilution, distilled water was used. PCR-amplified products were examined by electrophoresis using a 2% agarose gel.

### 2.4. Library Preparations and NGS

Adapter sequences suitable for Illumina NextSeq 550 next-generation sequencing platform were added to the primers (index primers), and an indexed polymerase chain reaction was established. The amplified PCR products were loaded on 2% agarose gel and checked for appropriate band size and negative control with a UV imaging system. After pooling the PCR products (triplicates) belonging to the same field study, their quality and quantity were measured with Qubit 3.0 Fluorometer (Invitrogen™; Thermo Fisher Scientific, Waltham, MA, USA) (Supplementary Table S2).

Sequencing libraries were generated using Nextera DNA Prep Library Prep Kit (Illumina, San Diego, CA, USA) following the manufacturer’s recommendations, and index codes were added. The samples were then sequenced on an Illumina NextSeq 550 platform using 2 × 50 bp PE chemistry. 

### 2.5. Bioinformatic Analysis

Bioinformatics analyses were completed through the Linux/Unix-based operating system terminal. The quality controls of the “.fastq” formatted forward and reverse read sequences obtained from the Illumina NextSeq 550 device were checked with the FASTQC program. Further analyses were performed using the “ObiTools” package (Supplementary Data S2). Sequences were aligned and merged with the code “illuminapairedend” with phred score threshold of ≥30. After that, filtering of non-merged reads (obigrep), trimming forward and reverse primers at both ends by allowing a maximum of 3 mismatches (tagcleaner), cleaning of duplicate data (obiuniq), and cleaning of unnecessary data from each sample header (obiannotate) were performed respectively. The expression ‘uniq’ was used in all sequence data obtained as a result of this workflow. After this step, two different filters were applied. In the first filtering, uniq sequences with more than 2 copies and longer than 100 bp were kept, and the abbreviation ‘c2l100’ was used for them. In the second filtering, uniq sequences obtained after the first filtering were cleared of PCR and sequencing errors and recorded as ‘c2l100clean’. For each primer and field sample, 3 different data were obtained as uniq, c2l100, and c2l100clean. All these files were matched with the online NCBI GenBank database using megablast tool of Geneious Prime. In addition to these, all fasta files were uploaded to SILVAngs. For further analysis, data that had ≥98% pairwise identity were kept [28,29,30] and sequences found in negative control reads were removed from the data. Principal component analysis (PCA) was executed using the prcomp function in R to study the structure of all locations and their relationship to the number of OTUs and abiotic variables. All statistics and visualizations were created using R (version 4.1.1, https://www.R-project.org/. Accessed on 11 May 2021) within R Studio (2021.09.0 Build 351, https://www.rstudio.com/. Accessed on 11 May 2021).

## 3. Results

The results of the physicochemical analysis of the thermal springs of the four fields were examined within the scope of this study and are indicated in Supplementary Table S1. In summary, it was hypothesized that Nevşehir-Kozaklı and Ankara-Kızılcahamam thermal springs host hyperthermophiles, Yozgat-Boğazlıyan thermal spring hosts thermophiles and sulfate/nitrate oxidizing bacteria, and Muğla-Dalaman thermal spring hosts halophiles and extremophiles. Based on these results, it was shown how the temperature or chemical content differences in thermal spring waters affect the microbiome. 

In the sequencing phase of this research, due to the use of Illumina NextSeq 550 and a 2 × 150 bp read size, the forward and reverse reads of the amplified sequences with 16SV3F-R primers (200 bp) were merged, and sequences amplified with primers 515F-806R (350 bp) completed were both merged and separated as forward and reverse.

**Figure 1:**
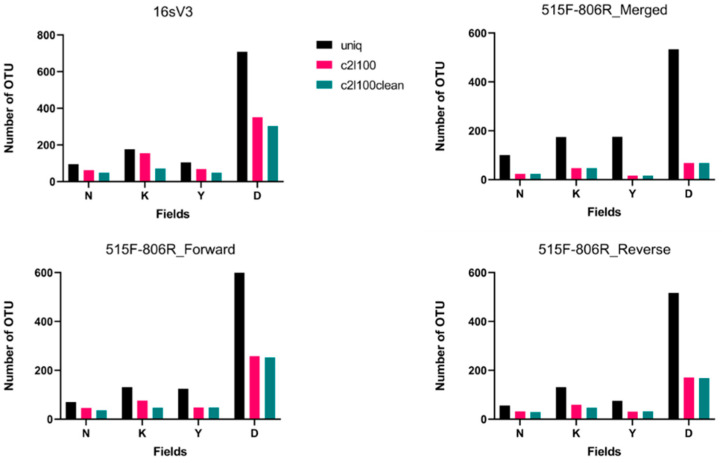
Number of OTUs detected in each fasta file. N, K, Y, and D represent Nevşehir-Kozaklı, Ankara-Kızılcahamam, Yozgat-Boğazlıyan, and Muğla-Dalaman thermal springs respectively. Different colors represent bioinformatic filtrations: black is uniq, red is c2l100, and green is c2l100clean. Results belong to the pool of triplicate PCR pools of 4 field samples (12 in total).

After the implementation of the pipeline composed of ObiTools and online blast, matchings had pairwise identity over than 98% were kept. In Nevşehir-Kozaklı and Ankara-Kızılcahamam springs, which were classified as extremely hot, OTUs identified as *Thermus aquaticus*, *Thermothrix azorensis*, *Thermus scotoductus*, *Hydrogenophilus thermoluteolus*, and *Caldimonas manganoxidans* were detected. In Yozgat-Boğazlıyan spring, OTUs identified as *Desulfurispora thermophila*, *Thermodesulfovibrio yellowstonii*, *Thermodesulfitimonas autotrophica*, and *Vulcaniibacterium thermophilum*; In Muğla-Dalaman spring, OTUs identified as *Hydrogenovibrio halophilus*, *Sulfurimonas crateris*, and *Desulfovibrio salexigens* were detected (all detected OTUs are shown in Supplementary Table S3). All individual OTUs for each field were counted. As a result of this analysis, the graphs shown in Figure 1 were drawn. 

As seen in Figure 1, filtration of the data means a decrease in the number of OTUs detected in each field. In addition, it was observed that there is a slight difference between c2l100 and c2l100clean data when comparing both of them with uniq data. Importance of the ‘c2l100’ comes from elimination of the singletons. Singletons are sequences that represented in the data only once. Since it is quite difficult to obtain a single read from a complex sample, mostly they are classified as sequencing errors [31]. When detected OTUs were investigated in detail, it would seem that content of the uniq data was not specific for special conditions of fields. For analyzing the data more accurately, additional filters were applied. With the c2l100 filter, results became more customized. However, the application of an additional clear filter caused data loss. This part of the study suggests that the c2l100 filter will obtain the best result. When merged and separately analyzed forward and reverse data belonging to 515F-806R primers were compared, it seems that the number of detected OTUs was lower in merged data. In addition to that, when both 16sV3 and 515F-806R primers were compared, 16sV3 showed more OTU matches. 

**Figure 2:**
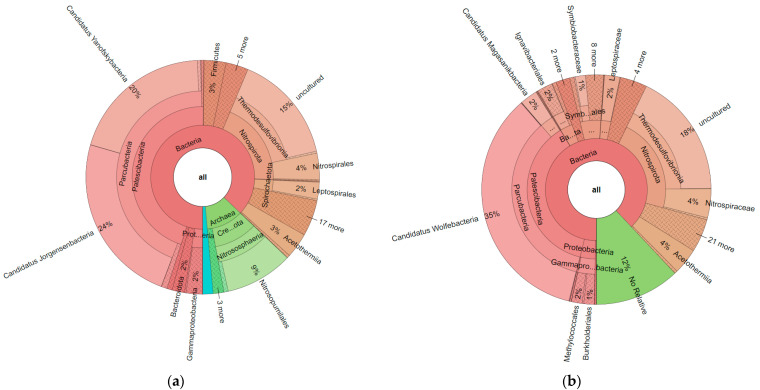
Krona chart results obtained from Yozgat-Boğazlıyan field for c2l100 filter; (**a**): 515F-806R_Reverse; (**b**) 16sV3. Results belong to the pool of triplicate PCR pools of 4 field samples (12 in total).

Fasta files obtained from ObiTools were uploaded to the SILVAngs online system. Based on the SILVA Reference alignment and taxonomy, each read was aligned, quality verified, and classified. Sequences in the SILVA reference dataset that did not have a closely related sequence were regarded as “unclassified” and allocated to the virtual taxonomic group ‘No Relative’. When all krona charts obtained from the SILVAngs system were compared (Supplementary Data S3), some critical points for the data analysis were observed. First of all, for 515F-806R primers, archaea matches were obtained, as seen in Figure 2. When these two krona charts were compared with each other, groups such as Thermodesulfovibrionia, Acetothermia, and Parcubacteria were commonly found with both primer pairs. However, it seemed that 16sV3 primer pairs only amplified bacteria, not archaea.

While thermal springs are considered as extreme environments and archaea’s most known characteristic is that it ‘can live under the extreme conditions’, data including archaea were investigated closely. It was observed that only in the uniq data of Yozgat-Boğazlıyan and Muğla-Dalaman field was Asgardarchaeota found, which is the first report for that group from Turkey. Odinarchaeia in Yozgat-Boğazlıyan and Lokiarchaeia and Heimdallarchaeia in Muğla-Dalaman were detected (Supplementary Data S3). 

For both analyses, online blast and SILVAngs, we showed that eDNA metabarcoding technique could be applied to detect extremophilic microorganisms in thermal springs, and not only laboratory investigations but also bioinformatic analysis can affect the results. For interpretation of the data, the SILVAngs system provides convenience but for species-level identification online blast should be used.

**Figure 3:**
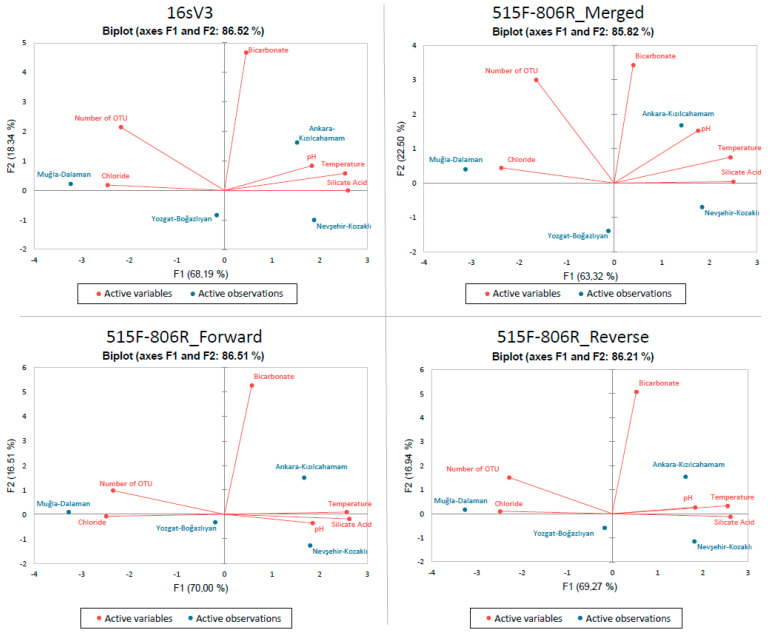
PCA of each online blast results obtained after c2l100 filtration. Fields are represented in blue color while physicochemical parameters and number of OTUs are represented with red. Results belong to the pool of triplicate PCR pools of 4 field samples (12 in total).

Principle Component Analysis (PCA) was applied to c2l100 data for both primers and it is represented in Figure 3. Physiochemical variables such as temperature, pH, bicarbonate, silicate acid, and chloride were compared with number of OTU data. 

With this analysis, it was observed that number of OTU is positively correlated with chloride while not correlated with bicarbonate and negatively correlated with temperature, pH, and silicate acid. When the results shown in Figure 3 were analyzed in terms of fields, there was no direct relation observed (Supplementary Table S4).

## 4. Discussion

Springs arise when groundwater emerges freely from the Earth’s subsurface in many rock fractures, eventually pooling to produce a stream-like movement. Physical environmental characteristics, particularly temperature, have long been recognized as key regulators of microbial diversity in the environment [32]. Temperature is a limiting factor for most aquatic organisms, affecting essential ecological processes such as primary productivity, decomposition, respiration, and the carbon cycle in communities [33,34,35,36]. Likewise, we found changes in OTU richness between springs of various temperatures in our findings. The primary taxonomic groups were discovered exclusively in relation to a specific temperature level in our investigation.

This study set out to develop a workflow to identify thermal spring microbiome with eDNA metabarcoding and next-generation sequencing methods for the first time in Turkey. For the determination of thermal springs, the temperature was the critical aspect. With four different water samples, two primers, three bioinformatic filters, and two databases, factors related to wet-lab and in-silico which contribute to microbiome identification were examined. The results of this investigation showed that springs with extremely high temperatures (89–90 °C) were dominated by hyperthermophiles; the spring with a high temperature (52 °C) was dominated by thermophiles, and the spring with a low temperature (26 °C) and high salinity was dominated by halophiles and sulfur-oxidizers. 

16sV3F-R and 515F-806R primers, which are frequently used in microbiome studies, are the primers that best reflect diversity in terms of the region they amplify (V3 and V4, respectively) [37,38]. While the 16sV3F-R primer pair-matched more genera in bacterial pairings, the 515F-806R primer pair obtained bacterial pairings at a rate that could be considered close to 16sV3 in most field samples, as well as pairing with archaea. When the results obtained with the 515F-806R primers were scrutinized in terms of the number of OTUs, the number of forward reads was higher than the reverse reads. The conclusion to be drawn from all these is that if the 16sV3F-R primer pair is decided to be used, archaea cannot be reached, and if the 515F-806R primer pair is used, the forward and reverse readings should be analyzed and interpreted separately. The matching step is also directly related to the database used. When we used the online blast, we could not reach any archaea but with SILVAngs we observed archaea groups. Thus, we can suggest that adding different databases can bring new insights.

It is possible that the variation in species spectra obtained by different 16S rRNA gene fragments is due to primer specificity and PCR errors [39]. One primer combination is more specific for particular taxa, while the other amplifies the genes of other taxa more effectively. There are two counter-arguments to this notion. First, lower primer specificity would result in a decrease in the abundance of specific taxa, disrupting distribution uniformity. Second, the two pools of major OTUs generated by two primer pairs contained different species. While some of the OTUs from the data collected with various primers matched the same full-length sequence from the SILVA database, others did not. The activities of these fragments may be related to the idiosyncrasies of bacterial taxonomy identification conducted using distinct 16S rRNA fragments.

In this research, we used two pipelines to detect the content of different thermal waters: ObiTools with online blast and SILVAngs. We can obtain matches at the species level for the online blast, while genus was the deepest taxonomic level for SILVAngs. Which pipeline will be used should be shaped according to the research question. Species-level research cannot be implemented with SILVAngs, as shown in Figure 2 and Figure 3. However, in our SILVAngs results, we saw that we have Asgardarchaeotas, which have been identified as Eukaryotes’ closest existing prokaryotic relatives [40] and it is the first report of Asgardarchaeotas from Turkey. It is reported that Asgardarchaeotas were identified in sulphate-rich [41] and hot hydrothermal cores [42], which are similar conditions as those in our Muğla-Dalaman and Yozgat-Boğazlıyan fields. Although they have been eliminated from our data after filtration related to low count-related filtration, we revealed that a more detailed study should be carried out regarding this evolutionarily important group. In comparison to prokaryotes, evidence suggests that eukaryotes have a poorer thermal tolerance [43]. This could be related to each group’s biological traits. Bacteria first appeared and evolved at a period when the earth was subjected to high temperatures [44], and there are today bacteria that can survive in extreme temperatures [43]. 

Each datum was analyzed with primer-filter-database combinations, which were 12 in total. Determination of the best pipeline for identifying microbiomes in waters with extreme conditions depends on many factors. In literature, we saw an unresolvedness due to the wide variety of analyses. This leads to us interpreting a new combination of currently available pipelines. From the amplified region of the 16S rRNA gene to the PCR-error cleaning tool used in the workflow, each step of the pipelines used in this research is open to modifications. Therefore, the effects of each modification are reflected in the results in a different way. 

Metabarcoding has become the gold standard for characterization of complex microbial communities linked with environmental samples over the last decade. Although this method may not be able to identify all taxa in a sample, the information obtained by a comprehensive metabarcoding technique is reliable enough to make appropriate biological inferences. However, strong methodologies and universally acknowledged standards are required to generate reliable and verifiable data, such as biodiversity estimations and taxonomy assignment [44]. Until now, most metabarcoding methods have depended on Illumina sequencing technology, which limits the length of amplicons to 600 bp. For many bacterial taxa, this is a significant constraint in terms of taxonomic resolution, as taxonomic assignment of short-reads at the species or even genus level is sometimes tricky.

Overall, in this work, we showed the difficulties of identifying extremophiles in thermal water samples and the effects of both wet-lab and in silico parameters. For the general and genus-level identification, analysis of filtered (c2l100) V3 and V4 regions’ amplicons on SILVAngs provided the best result, while for more detailed and species-level identifications, analysis of the same amplicons on online blast can be used.

## 5. Conclusions

Although more research is needed to disentangle the effect of different environmental factors affecting the microorganism community structure in the studied habitat, the significant variation in microbial diversity observed in the studied hot spring systems could be partly explained by the influence of temperature. Mutualistic connections appear to be important in the formation of stable microbial networks in the hot springs investigated, according to our findings. The significantly more sophisticated bacterial network outlined in our study could imply that bacteria’s more adaptable trophic tactics are favorable to their survival and fitness in harsh environments. The different extremophile microbial strains obtained in this study could be a valuable source for isolating new bioactive chemicals, and they certainly warrant further investigation. Across each spring, there was a substantial presence of taxonomically novel sequences. This indicates that these unusual thermal springs may include novel bacterial/archaeal communities and possibly a gene pool. To explore and harness the potential biotechnological and economic value of such bioresources, studies targeted to enhancing the cultivability of microorganisms are crucially needed.

The methods used for microbiome profiling vary at each step in the workflow. From sample collection to computational quantification of populations, it is critical to optimize each applied protocol and combine appropriate analyses. Since variations in both fields, and experimental and computational protocols are shaped by the environment to be studied, living groups, and technological opportunities, there are lots of difficulties to build a fixed workflow for ‘microbiome metabarcoding’. This work contributes to existing knowledge of microbiome detection from extreme environments by providing an optimized workflow. To our knowledge, this is the first study conducted on high-throughput sequencing of bacterial/archaeal diversity of thermal springs of Turkey and the first report of the existence of Asgardarchaeotas in the microbiota of Turkey.

## Data Availability

The NCBI BioProject database was used to store all of the raw data, with the BioProject ID PRJNA837878.

## Supplementary Materials

The following supporting information can be downloaded at: https://www.mdpi.com/article/10.3390/biology11070998/s1. Table S1. Results of physicochemical analysis; Data S1. DNA extraction protocol; Data S2. Codes used in bioinformatics analyses; Table S2. Results of Qubit measurements; Table S3. All OTUs detected by megablast in NCBI GenBank; Table S4. Raw data of PCA; Data S3. Krona charts obtained from the SILVAngs database.
