# Supplementary material for: Revealing the Microbiome of Four Different Thermal Springs in Turkey with Environmental DNA Metabarcoding
Source: Biology (Basel). 2022 Jun 30;11(7):998. doi: 10.3390/biology11070998 (PMC9311576; doi:10.3390/biology11070998)
Supplement: Supplementary file 1 [file biology-11-00998-s001.zip › Supplementary Data S1.pdf]

Supplementary Data S1. DNA extraction protocol

DNA Isolation from Sterivex filters with QIAGEN DNeasy Blood & Tissue Purification Kit

1. Remove inlet and outlet.

2. Prepare a lysis solution by mixing **450 µl ATL, 50 µl proteinase K ve 1500 µl distilled water** per filter.

3. Close the outlet cap and carefµlly add lysis solution. Pipet the solution between the outside of the filter and the capsµle walls. Close with an inlet cap, seal with parafilm. Handshake vigorously for a few seconds.

4. Incubate, while rotating, at 56°C for overnight.

5. Handshake filter capsµles vigorously 5 times.

6. Remove all the liquid from the inlet end of the capsµle. Insert into the capsµle to 5 mL tube and centrifuge at **5.500xg (4.200 rpm) for 1 minute.**

7. Vortex for a few seconds.

8. Add **200 µl AL** and incubated at 56°C for 10 minutes.

9. Add ice cold molecµlar grade 99% ethanol to the sample in equal volumes.

10. Shake vigorously.

11. Pipet the mixture (max 650 µL at a time) into a DNeasy Mini Spin column in a 2 mL collection tube provided in the kit.

12. Spin in micro-centrifuge preferably at **4°C at 6000xg (8000 rpm) for 1 min**.

13. Discard flow through.

14. Repeat steps 11-13 until all sample is filtered through DNeasy Mini spin column.

15. Place the DNeasy Mini spin column in a new 2 ml collection tube (provided), add **500 µl Buffer AW1**, and centrifuge **for 1 min at 6000xg (8000 rpm)**. Discard flow-through and collection tube.

16. Place the DNeasy Mini spin column in a new 2 ml collection tube (provided), add **500 µl Buffer AW2**, and centrifuge **for 3 min at 20.000xg (14.000 rpm)** to dry the DNeasy membrane. Discard flow-through and collection tube. Place spin column in a new collection tube, centrifuge **1 min at 17.000xg (13.000 rpm)**.

17. Transfer spin column to a new 1.5- or 2-mL tube with caps removed.

18. Add **50 µl AE** to spin column.

19. Incubate **for 10 minutes** at room temperature.

20. Centrifuge **for 1 min at 6000xg (8000 rpm).**

21. Discard the spin column.

22. Transfer DNA to pre-marked tube with lid intact.

23. Store at -20°C or at -80°C.
