# Supplementary material for: Revealing the Microbiome of Four Different Thermal Springs in Turkey with Environmental DNA Metabarcoding
Source: Biology (Basel). 2022 Jun 30;11(7):998. doi: 10.3390/biology11070998 (PMC9311576; doi:10.3390/biology11070998)
Supplement: Supplementary file 1 [file biology-11-00998-s001.zip › Supplementary Data S2.pdf]

Supplementary Data S2. Codes used in bioinformatics analyses

```
#For 16SV3F-R primers
#qc analysis
fastqc *1.fastq
fastqc *2.fastq

#alignment f/r
illuminapairedend --score-min=30 -r *2.fastq *1.fastq > filename.align.fastq

#filtering unpaired
obigrep -p 'mode!="joined"' filename.align.fastq > filename.merge.fastq

#removing primers
perl /mnt/d/kali/obi/tagcleaner-standalone-0.16/tagcleaner.pl -fastq filename.merge.fastq -out
filename.trim -tag5
ACTCCTACGGGAGGCAGCAGT -tag3 ACCGCGGCTGCTGGCA -mm5 3 -mm3 3 -info

#remove repetetive sequences
obiuniq -m sample filename.trim.fastq > filename.uniq.fasta

#removing unnecesarry values from header
obiannotate -k count -k merged_sample filename.uniq.fasta > $$ ; mv $$ filename.uniq.fasta

#filtering according to minimum length & count
obigrep -l 100 -p 'count>=2' filename.uniq.fasta > filename.c2.l100.fasta

#clean pcr/sequencing errors
obiclean -r 0.05  -H  filename.c2.l100.fasta > filename.c2.l100.clean.fasta

#For 515F-806R primers
#qc analysis
fastqc *1.fastq
fastqc *2.fastq

#removing primers with Trimmomatic via Galaxy Bioinformatics
#remove repetetive sequences
obiuniq -m sample filename_F.trim.fastq > filename_F.uniq.fasta
obiuniq -m sample filename_R.trim.fastq > filename_R.uniq.fasta

#removing unnecesarry values from header
obiannotate -k count -k sample filename_F.uniq.fasta > $$ ; mv $$ filename_F.uniq.fasta
obiannotate -k count -k sample filename_R.uniq.fasta > $$ ; mv $$ filename_R.uniq.fasta

#filtering according to minimum length & count
obigrep -l 100 -p 'count>=2' filename_F.uniq.fasta > filename_F.c2.l100.fasta
obigrep -l 100 -p 'count>=2' filename_R.uniq.fasta > filename_R.c2.l100.fasta

#clean pcr/sequencing errors
obiclean -r 0.05  -H  filename_F.c2.l100.fasta > filename_F.c2.l100.clean.fasta
obiclean -r 0.05  -H  filename_R.c2.l100.fasta > filename_R.c2.l100.clean.fasta
```
