# Supplementary material for: Revealing the Microbiome of Four Different Thermal Springs in Turkey with Environmental DNA Metabarcoding
Source: Biology (Basel). 2022 Jun 30;11(7):998. doi: 10.3390/biology11070998 (PMC9311576; doi:10.3390/biology11070998)
Supplement: Supplementary file 1 [file biology-11-00998-s001.zip › Supplementary Data S3/16sV3 c2l100 krona/16-c2-l100---ssu---krona----Total---sim_93---tax_silva---td_20.html]

Javascript must be enabled to view this page.

magnitude
magnitudeUnassigned

16-c2-l100---ssu---krona---16d.c2.l100----Total---sim\_93---tax\_silva---td\_20
16-c2-l100---ssu---krona---16k.c2.l100----Total---sim\_93---tax\_silva---td\_20
16-c2-l100---ssu---krona---16n.c2.l100----Total---sim\_93---tax\_silva---td\_20
16-c2-l100---ssu---krona---16ng.c2.l100----Total---sim\_93---tax\_silva---td\_20
16-c2-l100---ssu---krona---16y.c2.l100----Total---sim\_93---tax\_silva---td\_20

2114214166136511435213689

15772171641

32513

11

11

1

1

1

1

3

3

3

65

65

65
2

5

4

3

3

1

1

1

1

21

21

21

2095114089136501434412045

594824143

91922

91922

81922

12

7192

1

38292124

7

3292

2

2

1

292

2

29

8

94

2106

1

110

110

110

3

1

7

1219

131500

131500

1

19

19

19

19

19

1
5

3

1

1

1

1

27

1

48

4145

1687474

116122

25121

25121

121

1312

911

911

23

6

611

1

527352

8

8
6

1

1

5

5

5

1

377352

1

12

12

12

2

1

52

463622933020

103622559

103622559

103622559

103622559

292912422

2812415

1297

1297

1297

112

627

27

17

17

17

15

2

3171538587199657

34

34

34

221

221

18

7

196

196

2

2

1

1

1

1

144

12

1

1

1

11

20171355458

31

265364

4

4

225364

2

31

232

2

2

1

11

3

1

1

2

51

1

1

1

4

31

31

3

1

414327

414327

3

1

1

3

2

104

154322

2

1

4

163078

1

42

1

1

12

1

651

2

1

221

1

12

55

1

15

2

1

302

302

1

2

110721331

4

562

562

2

2

1

2

1926

126

1

1

2

2

10

2

8281

43

324

11

1

3

3

1

1

2
155

5

11

2

4

5

17

116

116

116

12

12

12

1

27

27

27

16144

6110

66

66

30

4

24

2

74

1

73

134

134
3

23

8

1

109146654614321

251

251

5

201

21

21

21

1

1

1

4

113

113

1

13

1

1

1

14164

14164

14164

231

231

231

1836571536

22

21

1

1

1

1

1

2

2

1

1

932768373

1297

829861373

1

1

4383113

1

2383113

1

17109312

16109312

16109312

1

1

12

9

2

3

4

3

3

1

1

1

1
5

1
4

3

37574555915

37304495013

19552

144250298

165399131

1

1132

2

15

27692

6

1

2781

1

1

2

2

3

3

3

2

1

19

19

122

1

1

1

22

2

2

2

1

1

74119923767

71118421161

5218421161

652

2

2

172

172

1

1

353

1

345

7

38

38

86

86

2

2

2

1

1

1

2

2

2

2

2

2

3155

272601

113

21

57

57
6

51

16

2011

2011

2011
91

4

2

51

816

620

35

2

2

2

2

871318

71297

71297

71297
1267

321

19

2

1

5

4

11

11

11

11

2

2

1

1

1

1

6610

6610

6610

1

32

31

1

2

4

14

78

11

1

3

126991038

1269910

1269910

1

1991

1991

49451

15091

3436

23921
8

52

902

84

11171

229

92

92

14

92

42

5

50475

74

4162

45573

38

38

38

38

11

11

11

31100

32

4783822175

5422187

1

7

7

7

1642

41
1642

4

1

12

14

14

14

317

31

10

12

1

1

1

6

7

7

5

86

8

8

6

6

39

6

4

2

1

1

4

8
29

2

10

5

4

14164

8164

1

1

1
3

2

4164

3164

1

6

386

28

28

26

1

1

106

333162
1

36
332162

18

278162

278162

318

312

1

81126938424450

11

1

1

1

1

110

3

3

17

16

1

1

18

77125138324426

1387915419213

1387915319012

1387915319012

121

21

1

2

2

2

5

5

5

1

81

81

11

7

1

1

1

2

2

2

3

3

3

1

59516145

4861185

2491122

2

411

335

2

955

32

53

2

4

1

1

1

1

1331

1

1

331

331

57236212283

5721922163

5544129

21751073

52

52

1618

1

8

16

11374

11374

1814

181

13

13

1

1

7768492233
1

121344

1

12

1137

4

1

1

1

1

5

5

5

13

27

484134171
1

183476

8
183476

5

23420

1

1132

9

6

3

4845
11847

72

1

44

333

333

333

91

91

11

1

11

11

11

36

1

1121

2

1

1126

131321

2

2

2

1

1

1313

1313

3

10

13

7

1

1

272

272

272

272
142

10

3

999965355142

10911344

22

5

8

308

133

273341

4

3

3

3218

2

1211

2

5

1351

1351

229
69439

8839

8

308

24

37

3413
17444344780

61224756

3

1

1

12

2

1

2

8

101

888

2

9

1

1

31

1

2

14

14

4

2

22

4

84

84

84

38230

58828814444396

2155

2155

265

9

9

11115303

19

10115294

1854

1854

1

2

329

691

691

170

49

49

49

45

4

55

5

5

5

5

5

5

8473

8473

513257944015

271037510

4103759

2

4

6

41

1

320

2

9

103

6

171

11

4

1

1

1

1

44

283946

23

13

10

292

1

1

92

1

1

3542

3542

3

2

1

4

33

106

81

25

3

1

1

1

1

1

1

5

27
1

7

11

1

7

9

6

1

1

1

11

5

5

1

131

10

31

11438541
2

1

673835

1

2

2

1

1

7

1

1

2

163834

4

11

23

1

3

1

422

1

1

1

32

3

292

26

1

22

732

432

3

1

1

1

19195

2

1

1

15195

11

195

3

1

2

1

1

245185

1

144

2

141
81

6

1

1

4

14

14

1

51

51

51

51

1

1

3

1

414

414

312

312

35

7

12

1
36689018357912797478

26557281010706333

192

192

1

191

32

32

32

84

1
84

83

6

5

2

3

1

1

70

70

1

69

334108507469614

322953822

5

1

12

3

167

1

153

12145302

1

13

3

1

6
2

4

13191

1

1

13171

3494122282

2

2

2262

327262185

924

1

3

11

1

1

1538025615224

316

7

1

113439173

36521313334

3

3

3

1

6

1

2

23

1

52

144025381

14

4

402528

51

1

2145

1

145

1

21

21

2

2

6771493195910

6771493195910

21271335

1874

214

125

716

448622

3

2

3

28

7458477

8

2

10

13

2

11

11

1068082083

266782063
52

7931

15131912

28

61

1629

7

661

1

1

3

1

61

21

1

1

1

83

83

2

2

61

4

4

4

12

12

12

2

2

2

5

3

3

2

541662

541662

1

1

3

3

2

1

3161

11

1

10

5

1

21

34

2

1

1

7

1

2

1

1

1

1

122

122

12

11

54702151

1757211

11

10

10

84

72

12

213

14

2

1

1

433

2

2

2

44407

49

4

9

3

3

12

7

5

74

1

12

3

2

2

2131

29

14

14

15

1

1

1

3

3

3

3

8

8

8

8

3391329025695734445

151

151

146

5

1

52

691373620
1

1

1

616

616

518

515

1

2

1212

1

212

4

1

3

286612

1

1

86512

1

1

1

5

4

1

5

5

12

6

6

3

3

332502

10

12

1

1

1

250

16

3

103

37

66

1663

1663
2

414

40

1188

3

16

1

1411

3
1411

19

2

2

7

1

362521

162521

12

263

171

345

2

1

1

3

3

3

59

4

78

46

12

4

30

32

2

2

1

13

4

3

6

1

3

2

2

1207

163

23

140

44

44

1

1

22

22

2

2

31

31

31

2991729522355201

12

12

3

1

2

6

3

1

2

584496174

3

2

32

146

181

4

351

251123

1

1

3

12

217745528

214

6121

13

1

21563

47

443111

241

44

1

1

5

1

173

173

21

19

6802387289

16

9

1

1

6

9

1

256

6110

4

5610

15

1

1

508296154

2

2

1133

1

2162

4128

4128

24792

2401

4391

1

1

30916235

30916224

1

1

1

1

2431

2431

242

11

72

72

72

1

1

222

222

222

3236294

3236294

1

23629

1

23

28

28

5

21

2

520

520

520

46

46

1

38

7

272327203952357

1

1

18

18

16123203328803

92

163

1

560203328713

1

1

5

1

1

6

6

75381

1

44

3138

3

1

1

1

2

2

3

3

2

2

16

14

2

83

83

1

1

14

14

8

8

26

26

3158423544

3158423544

22

2

2

55

55

1

10

39

5

1

21

3

3

3

3

15

1

7

2

2

3

5

5

3

2

10

1

1

1

1

2
1

1

1

1

3

3

3

1

1

1

1

4

12

12

12

12
1

2

7

2

12899691

12899691

1559

1

1

559

559

289411

289411

1

28941

97241481

97241481

236731

236731

2

36731

970475

320
970475

650475

1

52

7

1

6

6

6

45

45

3

5

2

6

2

13

2

11

1

12

1

1

1

1

12

47721493

1420

2
1420

319

11

1

1

8

8

31

81

3276

141

141

121

2

1113

1113

1

113

11

1

1

1

21

21

21

9165

512

1

1

42

22

2

2

2

1

2

3

334

1027325

1

52732

52732

1

121

121

121

121

1

21

682712

682712

682712

392712

2

32275

57

29

4

21

4

1

119

714

134541401190

3401

1

1

1

1

1

126

126

126

11

25

12867
1

1

2812

2812

2812

1

1

1

51

51

51

1713951

111

111

1

11

1389

651

1

1

1

4154

4154

151

2315

1

241

241
171

7

1

1

3

3
2

1

1

17

513

2

147

147

147

147

5063790394

312

312

72

47

25

11

3
110

15

72

2

5

13

119

22

94

3

448

448

368

308

3

1

2

1

1

7

7

1

1

1

1

4

4

4

2

2

3

3

3

3

2

2

1

1

1

895

5

5

5

845

7

5

1

1

17

1

1

7

2

1

5

1

3

1

2

523
1

2

14

62

1

1

11

11

9

6

42

1317

7

3790

3790

3790

208

3582

250

250

250

250

27

27

27

27

433

11

42

2
42

12

1

201

11

11

1

1

8

3

5

8

8

2

7
8

1

1

1

1

1

1

8
7

1

1

1

2

2

2

2

2

1

1

1

1

1

1
