# Supplementary material for: Revealing the Microbiome of Four Different Thermal Springs in Turkey with Environmental DNA Metabarcoding
Source: Biology (Basel). 2022 Jun 30;11(7):998. doi: 10.3390/biology11070998 (PMC9311576; doi:10.3390/biology11070998)
Supplement: Supplementary file 1 [file biology-11-00998-s001.zip › Supplementary Data S3/16sV3 c2l100clean krona/16-c2-l100-clean---ssu---krona----Total---sim_93---tax_silva---td_20.html]

Javascript must be enabled to view this page.

magnitude
magnitudeUnassigned

16-c2-l100-clean---ssu---krona---16d.c2.l100.clean----Total---sim\_93---tax\_silva---td\_20
16-c2-l100-clean---ssu---krona---16k.c2.l100.clean----Total---sim\_93---tax\_silva---td\_20
16-c2-l100-clean---ssu---krona---16n.c2.l100.clean----Total---sim\_93---tax\_silva---td\_20
16-c2-l100-clean---ssu---krona---16ng.c2.l100.clean----Total---sim\_93---tax\_silva---td\_20
16-c2-l100-clean---ssu---krona---16y.c2.l100.clean----Total---sim\_93---tax\_silva---td\_20

57511548193018372042

1372017302

55801523192918291737

36

314

1
7711182137

2

116

487895
1

18852

18852
4

2814

1118

6

9

1

5

71

71

11

44

10

3

1

1

33

33

33

11

11

11

6419
13421

72

1

1

1

5

12826

2

2

2

1

12

1

4

1

1

12

187

13

1

1

121

13221

1

1

132

132

3

2

10

2

2

2

55

414

414

312

312

35

7

12

16

454102031

263

16

16

1

1

14

103

39

8
29

4

8

5

4

6

2

1

1

4

4

449164

5

5

5

63

6

6

3

3

1

12

12

12

2

257

7

7

25

1

12

1

1

6

4

642

41
642

3

3

1

1414

814

3
1

2

1

1

414

1

314

6

33124
1

36
33024

18

27624

27624

9

9

9

1
9

2

4

2

1761284

621162

1

371162

1

14

6
14

6

1

1

9

9

9

114122

911

911

1

24

6

601

23121

23121

1312

101

1

1

1

151

1223419337

335

272171

21

16

113

14

6
14

8

923117161

2

2

2

2

2

2

2

2

2

54

2

2

4

1

3

12

12

30

3

1

26

5

5

1

1

293117161

1

1

1

2

2

2

107314

16

16

16

16

1

21

5736

5736

1

1

11310241

11310241

13941

13941

1394

1

184

1

1

84

84

52

7

6

6

6

1

45

45

2

1

3

12

13

2

11

2

1

5

6

443333469

17

1033266

1033266

1033266

1033266

66

2731390

133

133

133

261387

51

51

51

51

1

3

1

1

32

11

11

11

11

11

13942338

422

422

1

1

1

1

1

1

61171

31

31

3

1

351

111

1367
1

312

312

312

51

51

51

1

1

1

1

1

1

16

16

16

11

5

331

64

1

2

2

2

2

31411

31411

31411

16

4

8

4

15411

2

645

76

2

9

9

9

2

7

376421110255

114232

104230

2

33

17

66

66

1124

1124

1

12

849

849

3273199810

1511

1111

3

1

11

7

2

1

1

2

1

1

9212520
2

1

1

422

32

1

1

1

1

712

412

3

3

641234

2

151233

2

4

1

1

21

3

2

1

1

11

1

1

7

1

102

1

22

7

15

155246

10

6

4

3

3

1

2

1

1

1

1

11

1

1

222

1

1

22

1

25

5

3

2542

2542

24
1

4

11

1

7

1

1

81

31

5

5

5

9

1

6

1

1

18

9

9

1

213145

4

1

10

1

1

144

12

111
51

6

2

273585

6

171

4

1

11

1

1

43584

4

4

2

5

3

6

41

2

2

1

254

254

3

3

224

42

4

4

4

2

2

2

24

24

24

20

4

22

349820339

1312

4

4

4

2

2

26

26

26

26

3

3

3

3

820

820

820

793

27

433

42

42
2

1

12

11

7
8

1

201

1

1

8

8

2

11

11

8

6

2

2

2

1

1

1

1

1

1

1

152

152

46

21

2

5

13

5

11

58

18

3

37

37

24

13

7

895

845

17

5

4

5

1

1

1

42

1

3

1

2

1
523

1

2

62

1

11

6

14

9

11

7

1

5

1

5

5

5

27

27

27

27

472

472

7

7

392

3

1

2

332

1

1

116

116

116

116

27144515939

1

1

17

11

1

1

1

1

714

71

13

13

23137505918

38310145

3839124

3839124

121

1

21

3

3

3

2

2

2

2

2

2

3

3

3

61

61

5

11

132831243

1114

1114

912

2

11

8

4

4

131815183

2127103

11688

5128135

1

1

47375

34

2

211

22322

2

555

32

2

23

3

1

1

1

1

1

1

1

181

81

81

1

1

18

3

3

15

1

1

14

52

272227136129193

7

7

2

2

3

3

3

2

1

3

1

1

1

2

1

2

2

1

1

1

1

334

334

334

122

22

2

2

2

1

1

1

1

1

31

31

25

25

3

3

802121248321

1

1

1

1423312

1

1

1323312

1323312

4

17

17

1

7

5
1

1
4

3

111

111

61

5

1

1

1

23

23

23

1

1

1

3117943515

3112903213

8

1

632

2

14719188

12252

277161

5432

4

1

1

521

184515236

1

1

1

1

9321273

823573

197

1

1

22

21

1

1

1

4133113

1

2133113

1

2

2

9

9

9

14114

14114

14114

12

9

2

4

3

3

3

1

1661

115

115
3

8

1

4

646

17

11

2

4

64

64

25

1

24

20

3

3

3

1851464136

31

94221312

1

1

4

392

392

2

2

22

8281

324

11

1

43

197

1

2

1

27

9

2

2

1

2
145

2

4

5

1

3

11

3

3

3

16178

42

1

1

12

1

12

12

651

12

1

1

221

2

55

1

2

15

1

1

2

1

263364

223364

2

1

31

2

1

2

532

2

1

11

1

3

31

1

1

1

4

4

41514

41514

104

4

1

2

1659

3

1

2

2

1

14

14

14

9

9

9

31

31

3

1

3183

3183

1

2011

2011

2011
91

2

4

51

1

12

21

1

1

1

8

78165

4

5

5710

5710

5710

2

1

14

11

1

1

23

4

31

78

6

6

6

6

3

2

2

1

1

1

1

7149

7149

128
7149

1

316

2

15

420

47721424

914

512

42

2

22

2

2

1

1

1

1412

1412
2

11

1

1

8

8

311

3276

1113

1113

113

1

11

1

1

1

141

141

2

121

21

21

21

2

334

3

81

31

7
8

1

1

1

11

11

11

285

11

71

31
71

4

1

1

1

3

3
2

1

1

11018644411362109
1

8

8

8

8

24854616079628

32

32

32

23

2
23

21

3

3

3

13

11

11

2

53

53

2

31

2

4467

212

12

5

7

44

4

4

74

2

12

3

1

2

3

3

4

4

4

12

12

1

11

3

3

3

10974343

2904323
52

15962

241

32

179

14121

21

1

1

1

1

661

1

3

61

1

1

45142151

1713211

2

1

1

84

12

72

21

1

1

14

11

676661787

676661787

34072

1

47

2

2

154852

13873

28

9

11

14

3

25

3

373

4

2

2

2

49362

49362
2

2

1

1

21

1

1

1

3

44

1

1

3

12

11

1

331

1

2

1

2

3

1

5

5

3

2

433

2

2

2

8

1

1

7

4

3

29

15

14

14

1

1

1

2

2

12

12

1

11

333418850512

3185502

1

3

12

1

32

115533

110

1

1

13

3

1

21

21

2
6

4

1371

1

1361

1

235

1

35

1

15305212042

12

5

3

79515

2112

12

3

2

5

2

285131712

1

2

4

1

140611

4

1

4051

1

51

314191762

12

3

2

2

1

866

97

364145

3

3

3

3

84131828156681

3

3

3

23724418
1

1

1

1

1

23410

1

3310

1

1

12

6

6

2

2

2

2

4

1

3

24

5

19

5

1

4

18162

5

12

16

1

3

1

6

1

112

1

12

157

154

1

2

5

5

64

64

2

34121

14121

23

21

12

16

2

1

1

13

13

5

8

2111

2111

11

210

2
1

1

1

4

12

57

34

3

1

2

3

10

9

4

2

23

4

9

10

1

117

1

1

11

8

3

6

6

72

72

72

1

1

11

11

8

1

2

31

31

31

3

3

3

52

3

1

2

2

22

22

2

2

1

1

1

1

75176969639

2492

271

421

53

53

1

1

249105

24994

1

1

23412619

1

32

4

21

19

3

1

39

12

2

9110

443

443

12

12

23111

1

1

2

1

241

5

1

1

2561526

2363

3

7

212

1

321

6865351

593117

3

1

1

1

11

2

2

1

2162

1

2

1

1

4

112

84

117

18

4

3

2

1

21

9

4

3

2

11

216

216

216

1

15

11

4

2

2

2

1

3

3

1

1

1

1

58

58

58

14

14

2

9

3

144

144
3

2

1

7

2

12

101511794027

1

1

5

5

2

2

1571

3

1

127

16

16

14161761923

5121761873

1

1

1

1

72

14

5

5

11

11

1

1

3

3

1

1

7

1

1

5

2

2

2

2

32312094

32312094

3

3

12

2

10

5

5

25

25

5

9

1

10

217

2
217

14

3

8

53

137

1

1

1

1

5

5

2

3

32674

32674

267

23

1

1

1

1

1

74

810

3

3115

773161

773161

22611

22611

2611

2

7555

7555
23

5255

5
1

1

1

1

1

3

12

12

12

2

1

1

5942427

382221

3

1

92

16

16

16

7

7

8

29

322

1

2

2

22

2

2

1

126

9222

9222

1

8222

722

12

1

6422338272

3

3

174137228
3413

6

84

84

84

1

2

4

632204

31

8

1

2

1

9

12

1

14

2

3823

14

4

1

2

3

828

2

101

1

3210

2

124

4

2

1331

1331

101634

20

27331

223

8

6

133

5

124
3452

37

492

103

24

8

252

252

252

252
142

3

8

1435104

143510

143510

20

20

92

5

42

5081

391

1171

571

571

5415

502

18

4733

1

17

8
2821

118

11

6

38

1011

4

4

4

4

11

32513

3

3

11

11

1

1

1

1

21

21

21

3

3

3

1

1

1

1

65

65

2
65

5

4

2

2

2

2

2

1

1

1

1

1

1
