# Supplementary material for: Revealing the Microbiome of Four Different Thermal Springs in Turkey with Environmental DNA Metabarcoding
Source: Biology (Basel). 2022 Jun 30;11(7):998. doi: 10.3390/biology11070998 (PMC9311576; doi:10.3390/biology11070998)
Supplement: Supplementary file 1 [file biology-11-00998-s001.zip › Supplementary Data S3/16sV3 uniq krona/16-uniq---ssu---krona----Total---sim_93---tax_silva---td_20.html]

Javascript must be enabled to view this page.

magnitude
magnitudeUnassigned

16-uniq---ssu---krona---16d.uniq----Total---sim\_93---tax\_silva---td\_20
16-uniq---ssu---krona---16k.uniq----Total---sim\_93---tax\_silva---td\_20
16-uniq---ssu---krona---16n.uniq----Total---sim\_93---tax\_silva---td\_20
16-uniq---ssu---krona---16ng.uniq----Total---sim\_93---tax\_silva---td\_20
16-uniq---ssu---krona---16y.uniq----Total---sim\_93---tax\_silva---td\_20

132688850771226839878491690

3092539

65

65

5

5

5

1

3

2

23

23

1

1

1

1

1

762

762

762
211

43

111

1

1

2

2

2

2

2

1

1

1

438

438

438

141

141

141

141

182

179

179

3

3
128551821281075999638942862

1

1

1

1

7

7

7

7

7182821334200

71828213342

13

13

2

1

1

71827210342

5

2763123101

6838341

2079326

3

3

154

3

3

285
1443634

280

467

576621

3

630913

1326

588

588

4591

1

129

33

1

1

1

129832

129832

1

1

32

2762919

22122

2498117

436

198

198

198

198

4376182329226172058

398

398

396

32

2

362

2

2

380014772332583105

3086911235

3

1

24

863

7

1862
2172

31

5

152

5

32

1

1

5

2

87

1

10

662333

9

1369535

13

3

1

62

2

32

1

4

4

13

1

8

4

2

2

98953

1

22

67

1

1951

1

7

1

1

1

10

9

1

7

17685163213066

1885163212862

5

162

57

2

8

34

5

11638

1327

2

38516362

41

11624

8

13

20

8321

4

1

348

792293462944
27

30125124

2

25

113

31

41

182524

29

1
6818

7

1

33

1418

12

17

17

5712922152
3

2

15

1

56

15

2

2

1

1152912150

206

30

1

3

12

11

1

1

1

6

9

22

7

5

36

2

3

3

12

49212

6

17

20212

1

4

1

1

5

2028772442

17

1

1

15

1437

2

597

1

2

79

65

49

2

36

4

7

10

157615

1

47610

2

85

243

108

1

134

18
3

15

2

2

37

37

3

3

219

8

1

6

1

13

13

59

4

55

1651
1

451

63

52

4

6381

2111

426

1

2782411

2782411

2

65120591575

58120591471

753

3975940

3975940

1

123246

2

44432

44432

7

7104

6596

196

196

96

1

64

64

8

56

413043

413043

84

84

44643

4434335

4434335

1150102589755

1150102589755

11182323915

8
11182323915

1142304215

14

189

499226584

499226584
1547

13

425

342826334

171835834795

171835834795

1632179

232179

232179

14

14

118355135

118355135

5

11835513

1

1

28139

59
51

8

8

8
7

1

2180

28140

19118

644134102

644134102

644134102

262

197

7

18

40

382134102

28713428

16

3

26

5074

3

3

3

3

3

1
235925180026028885882943

14

14

14

14

185434495502043496235

2122

216

216

16

224

2

2

2

2114

1
2114

1714

3

1

1

1

3

3

3

6439079298010

65

105

2

6

25

462013

1

26

3

12

1

11

1911

31

838229296210
93116

68741326

12387216853

197455

38362121

437562

241

41

20

20

1

1

1

1
18913

8913

1

1

2

1

8710

29

29

29

249

249

242

7

8511271

8511271

727

91

115

3115

1

1

198

109

109

89

37348923541327570

37348923541327570

12125

11

3133

10

18211007791

2747

11

2

143055280

3234127

3

151530981

1717144

6

2124167738691

175

22762

114420

4

15171097512

1494

112

7204031338943

13

13

13

269253351

3
3872

272

4

29

1021812

4

4

65281
5

20

5

11

47

1

12

23

14

1

51

21

30

95

2

1

14

6

2613

3

3

2313

161

5

11

12

611

2

2

311

1

1

22825825253928072128

2

103412991459413
36

13

2

15

914715114063

111

413

12

115

55161

2

310

1

112186

192781142

1

1

1

861761291718

31

105717

1

1

117512817

39

84

11

11

11

183191483

69

63191373

62

11
21124656186825

210603413653

1

13331

465

1

123145

2

911183472

211

17

1

219

121

1411

11

3

1141
8624035403850532

3

3

43

23

13

124

19156

34

2

130

5

24515

411

12

1

217

121

1223113111731731

105362497841

695393

1

24

1

22816

3220

33

23858

4416

34

11

844

1

1

213

3

1

2

4

64

64

3232

1

30

222

8
521

2

381

2

2

6

201081272

8107272

611

2

4

21512213

447112367

21
447112367

41

1

5

3

30

2

65

1

5

10031

3

3

8

1

1

2

62

18

1

3

1

1

5

10

1

1

71

5

42

33

2

1

3

41

265

22

11

2714

1

1110715

38

38

28

1

3133

1

1

2133

2132

1

474

3
474

471

14111
1

111

3

1

1

1

10

78012

1

1

68012

1

1012

570

3161

3161

3161

34259433874

38118

2012

1716

1

71

71

861078

10

2

2

6

1214742660

14

11

14109

36

36

36

36

216871730521008450922708

6

401632

401632

7

331632

1

11

30179

30179

30179

76100

21
76100

7

40

361

5

39

1

1

1

2

1

3

5

9
16

5

1

4

2

2

15

9411

9411

9411

12

12

1

2

2

2

2

17699608292323021218

1

1

25

2

1

4

16740

16740

7113

1426740776

6432284

8

3

12611

172

127

3

21110

1

52401

32

161

140

3

8

1
53415732338911

154110

2514297

237344599

516

318

5

71

120311

96

1

10

131

2

1

1

52

3080

111

5

4

1

1

1

4151614320228

22

9

213

22

1

2150214219726

103246729615105
3

842

221639

378276

115

1009124148

1

2452

464541203218109
2

125

11

3

16

11

2

4

1

971

121

71328

43903

61

825

117

32058

1065

7

1

181735

1

13

143

1

12

5

21

1328

30171339876

121126101

4

1

34187101

5

4

25187101

112435511

3

619956

1

2

26

122075

1

1310

2112165

4113

2

9215

8

10

10

11

21682254180074169327

4

4

2231

2111

1

11

134

134

53

6

3

5

39

14

14

2119
7

13

19

1

5241

5241

49829610

210293

285

1

2

10

3

79

79

30960481753911

30960481753911

86

86

2

14

14

230

9

9

2

16

194

14

14

133

133

18

18

8

8

1

2
41

1

11

157918179402414216

1

11213

31520179392408510

11

2316

1

1

31

7397

11

91

91

91

1028

1028

28

4

4

2

101

101

101

3

4

7

7

7

731

731

31

700

8

42130

2029881127

102987117
2

2

1

21

59

1716

1148

1227

111

91

4

1

1

1

1

2

2

3

11

11

3783

3783

3203

54

4

1

6751

2691

8

1

8

46

1

1

1

1

52

1021

15

33

406

45

123

238

9387

9387
19

19

154

132

6489

2574

2

2

32

32

25

1

1

5

1445118610

1445118610

1

1

47

451186

3

8

162

2

2

16

16

13

303088

303088

1

348

26304

1

1

50542002491445
4

7
1

6

380822

7

16

5

378022

1

1

9

9

5

5

40187

1

39187

6122

2

612

1

1

2

2

46

44

2

5

2

3

697

184

513

3

2

1

1054649895

21

54245895

4

14

2

13

2

2

41187

4

1187

78

33

44

1

626

224

2

4

1

1

4

2

1

1

261114717

126

1147

1117

6

5

88

2

11

1

9

2

261

261

48

205

8

75

2

1367

257

137

12

9

9

102

35

11

19

2

35

5213

10

4

3

1

6

3

1

1

1

20

445

445

2

47

323

73

1

201229

4918

1

688

3230

161

4

1

11

2

2

3

3

310

310

21

2

2

191

91

1

1

3

3

3

3

3

3

3

2

16

16

16

16

2

6

8

38264323312655

811

811

811

211

6

22

22

5

17

17

41

41

41

30

2

4

5

103

103

1

93

52

11

3

30014

41
42

1

1504

7

2

2

73

3

62

8

1

44

44

60
4

56

3

3

5210

14
5210

11

8

810

2

9

56

20273

20273

20273

20273

23454

23454

15
797

125

2

515

5

6

101

1

27

90

6234

4

424

180

19

835

473

362

1672

1672

1672

1672

3402092

3402092

4

4

2902092
1

18

2402092

26

5

4

4

3

2

1

39

39

56

64218

59617
3

3238
1

38

6

1

93

564

32

10

751

93

2

30

18

4

8

329

40

3

36

1

160
1

6

20

7

2

33

7

7

77

8

461

461

461

43213

43213

43213

1283

41863

67

2612168

1

1

1

1

1

277

277

277

5616

271462

62148

23

23

11

11

11

12

198

198

198

3

3

177

18

129510

129510

635
129510

152

4

13

348

2379

37
4

14

14

14

2

8

4

1

18

526772818311090361

3

2063

7768123

75114

489

4

1

3

89

31

31

478765218231087182

316951389826

2

2

6

6

61124532

49

1

1

1

1

11

26216

7

2356

9

1

22

22

1

1

13

13

22

2

2

1

1

20582935719

1742

5

41

11503110

225893188

6

26

11313

11313

212

1

110

1

3624

3624
1

13

2

62

1127213

927213

927213

2

2

212

12

12

1

1

1

110537061571096

121086

7

1176

2

21

109536860570290
1

1

1

12

1

57

107536060470183

22611

1

1

11

11

1

1

1

511

4

1

11

16

22

22

22

59181

59181

59181

3181131104624428

308

308

31798515812828

9667713318

308318879510

1331090

96

86

37104

11257026

11257026

1

1

2

2

2

6183

317

22
2

2

115

3166

3159

7

7

1215

23

911

21

21

1

1

1

1

51

5

1

11

32

208

3

2977

9

9

9

11836138833

770927

6465

6465

8

4405

164

34

124922

124922

592

6592

4136129626

4

57

57

57

59

59
35

4

3

17

9

2846129626

61

61

61

7
61

4

20

11

19

10

678

678

678

678

678

13819728205

411972827

2

2

2

113

381972814

9

42

18

12

3825

16151

16151

16151

16151

6560682452404340

11823992333138
249543

567062

103

36

5522

5522

5522

11

15

681

516

1

13

522

5

149

1

12

1

1010

21

61

50

60

15

282

324

28

2962

4

21172940

37

13

24116225

2

142

314

14441
444417511

37

262

198

1934

5691751

4

1

2626207

72592

13

31103

112

1

1

766762993

37

227501

56

140

174262991

56

761

91454

91454
77434

3

2

92

4

52

2146

2146

2146

2146
876

21

94

9

3

38136

4272921840881

106172

1
26616127709

16113

16113

16113

22555

39

17

36161261

6

191

191

2

9

3

6

16126

161

26

52

26

6

6

4

2

211

5421

40

551311813

551311813

541311813

118

5213113

1

1

369

27

21

21

21

6

342

342

38

10

77

28

92

75

16

1

29

3

48

8

2

7

3829122017742245

1411624

365111041648615

7

7

4

2

1

8

8

8

2

2

2

82

22

21

1

6

6

9

9

8

1

16

1

1

2

2

13

13

23

23

16

7

32244111

2

1

1

121

9

1

1

11

42

1

2

2

1

4311

4311

5

2

3

171

17

1

2

2

9

1657

1534

2

18

103

856

6

832

17

1

218

218

13

3

10

1

35511001638510

164133366

1071

111355
91355

2

2

4421

2310

265
2310

245

3179

3179

714
923

29

2256

2256

639

1514

12

1

2

183721

3111

3111

3111

3111

7

12

16

3

4

72311214

1782

1782
1162

62

452164

114

114

1

1144

134
1144

110

110

4480395238421

27072

8672

184

184

2

8

3

171

47826520092

12062430

12062430
1246

128

24

78

5

11110

2

9

1

2

1

1

4

110

110

8559

79

79

5

159

59

1

108

108

108

26866

263

4

4

4

52

115

84

566

1

2

1

166

62

15713038

6813038
1

1
3112837

7

1

2

2012837

9

5
162

112

1

1

11
1

2

6

2

89

2941

2101
36

12

3

37

4
3

1

2

65

511

10

39

35

22

13
1

2

8

2

3281256
3

3278256
297

2789256

2789256

192

1
7223785

553637

553637

2401
553637

1486

20148

2

13

18

191

191

191

191

18

10

58657

58657

58657

6944

2

97

24

307

2

35

255

14

48

7

32

19

19

5

5

2

12

12

21

3531739132816574

6

7131

35159

2524132312993

313160

313160

313160

24941312932

1

1

1

53173553291

53173553291

53173553291

53173553291

2

66138234181707
7

4
1735133328

62169

29
210

1

5

1

2

2

2

151

963
1

63

63

8

93

11

2

209

22

242

19

1

1

16513171

198

84

3432221821200
6

6249

3

28

9

5211

5211

2423

114

75

75

75

8642321
3942305

4716

133181570

133181570
71

8

787320

40

417488

1

50

43

14

151803

151803

151803

41044

6

142

14

2

2

2

15

1

570449

1

11467259

25

25

25

112673

3

3

11267

17

167

94

26

21

5

25369616347015264007

1633

1531511237444267

84074178461

3

3

118133

6218

1

2

26

1525

4

3

5

3

6
127136

11

2

23

82

2

7

44

148

1221

48

1

14

14

7

1673

1

1

1

12

12

7017474

1

2

301718

5

2913

1164

1

1

24

17

1

7

44317

44317

1

1

1

91

91

91

3211

117

117

117

12

1

1

25352135111

56

51

5

19752135111

2

30277

2

1

13

1

10

22

5

5

3

1

1

1

1

1

1

5

2

1

30

5

13

1

7528

13

12

1610

23

3

8

143

73

73

13

6

8721843756

331123

42

4

2

811

2

692

71

12

3

1

2

5

2712313

21

1

26

1

93

1

463

667

15

1

18118

18118

22119

2

6

1

6

311

418

17

8128131

8128131

612871

26

1

1

1

2902552718

15

13

2

2902552703

6

24

71361

1

4

611

1

24

2

1

812532262

7

10

151

43

1

3512326

202126

1321

1317

4

424

24

3

1

3

2

1

2

1512

1512

1

122

3

11274

11274

1146

1070

1070

58

276

74

1

1

1

21

21

21

2158

2158

1148

110

31

42

15

20227

20227

1

1

18226

2

153

153

153

5

103

2426241235

2

2

2

424318

28
424318

42

84

4206

62917

6291

6291

517

514

1

2

309

32

36

241

3

3

3

3

925906131641053128

4

4

4

1216

1216

1216

974

13

1

10

2

2
844

53

4

15

1

9

4

15966137876

7

7

15266137876

1

1

15066137876

1131

1131

131

1

17
58

41
16

25

127225348639546

2

2

715

6

79

514

514

6

1

3

2

2

2

93192044928931

76164541428531

17275354

6

6

8333377515

4

2

1

1

4333377115

20

6

6

6

21

21

21

147148231

147148231

147148231

10111

10111

1

1

5

4110

314883247240075

119264268

4

1

631

16

192194

4
304691240837467

11

1

6

14

1149117

4

10266342220052

732

2

87

93

5755525

1

4101619741053

193

193

193

2102

2102

19

3

1882

8

125

3

3

1

1

120

14

14

120

120

9

9

11

11

1

11

11

11

1

1

376

3

3

3

3

1

1

1

130

4

964

1341

2

112

112

112

12

1

172

11332

11332

11332

11332
10327

15

12

4749812115531

8

3813

74146

74146
7

36

36

3
193

3

7

9

12143

2

21

1788319

2223126436

32223

1

2

2

2

2

223

223

2

2

2

10377

1
10377

10317

5

18121223

18121223

721223

4

1

1

3

3

1113

3

3059224

381621

1

416

3021

1921

2

421

1

6

6

11

2

1

1

1

6013

1183772686781111
2

6

3

73

4

4

1

3

31

31

11

1

1

2

440

440
3

137

3

4176906286

32190596

19821

19821

2

1981

57305

6

1

411717

411717

2

13
41935074

12

113

34

34

111

1190926

1190926

1190926

17

362

362

362

1

3

137259

137259

137259

71

7254

64

231754

63

4914544

176112682

176112682

61

12

12

12

12

12

10

7

7
4

1

2

2

1

1

1

1

1

1

1

2

2

2

3816294715084239048789
