# Supplementary material for: Revealing the Microbiome of Four Different Thermal Springs in Turkey with Environmental DNA Metabarcoding
Source: Biology (Basel). 2022 Jun 30;11(7):998. doi: 10.3390/biology11070998 (PMC9311576; doi:10.3390/biology11070998)
Supplement: Supplementary file 1 [file biology-11-00998-s001.zip › Supplementary Data S3/515-805_Reverse uniq krona/515r-uniq---ssu---krona----Total---sim_93---tax_silva---td_20.html]

Javascript must be enabled to view this page.

magnitude
magnitudeUnassigned

515r-uniq---ssu---krona---515dr.uniq----Total---sim\_93---tax\_silva---td\_20
515r-uniq---ssu---krona---515kr.uniq----Total---sim\_93---tax\_silva---td\_20
515r-uniq---ssu---krona---515ngr.uniq----Total---sim\_93---tax\_silva---td\_20
515r-uniq---ssu---krona---515nr.uniq----Total---sim\_93---tax\_silva---td\_20
515r-uniq---ssu---krona---515yr.uniq----Total---sim\_93---tax\_silva---td\_20

186101158060129051100389147094

43631095226814515742

602103674112512975

27876084112940

24

24

21

2

1

1

1

416318

416318
3610

1

11568

5

262919

2383314112370

2383314112370
121121

252

51115

403304012329

3

1

3823541

3823541

3700541

123

32428

2759125

2759

2759

2759

83

83

83

42

42

42

7

7

7

7

322626

322626

322626
242626

8

315

315

315

2070

2070

2070

48

3

1

8

301758520

301758520

23709
301758520

38

243

11210

11

10110

162

925851

2720

20

20

20

19

1

1

1

7

7

5

5

1

1

1

1

19

19

6

6

12

12

1

1

1392273

1392273

462

2

71

4

1
21

2

1

1

3

81

227

227

227

562

562

562

562

1476

1476

1476

1476

1476

3327

1

1

1

1

226

226

24

24

22

1

1

2

301

301

271

3

3

255

19

236

232
236

4

17383614173812531697162117993
1

1

6

6

6

6

46743152

5942755

5942755

32417
5942755

5117

33218

3

3

123

31

15

351114

351114

351114

182

44

27

8495

1

29

2

2514

40

813

12

12

4

4

1

7

7

2283

2283

2283

2283

4

24

9

29211

34

2

32

32

3

29

25811

25811

5

43

1

42

15

38

1

36

281

62

231

7

11291

291

291

291

291

11

1

17

164

21

10

43

43

43

43

46

442832392513533228

331734

331734

317

317

334

7679221710

7679221710

772262

1
712149

273

273

1138

5949

31

5918

1

3

41

1

888

6113

70198

70

70

70

198

198

198

40191932249253220

12418957

4

4

73

2

2

1

5

8

1

7

2

3

3

281895
1

41894

22

1

1

37

1

32

4

354

11

3

20

14

81637126
1

527373

7

82372

144

10

2

1

2

1

1

2

3

2

5

10

2

19

212

1

13

1

3

41

13

13

8

151

2

1

41

5

2

1

27

3

6

4

14

1121

113121
112

55

45

11

9

2

186

3351955330

7

22

15

1

1

126728

2181
2571

39

21112

1

13532
3

6

53

3

12

4

2349471

14610
31

1

3

2

318

83

1

15

8

5

73

36

1

11

25

255

651

3057

21

597

223

2

36916

36916

37

92

92

1

759

1

243

515

34
72

29

4

5

26

30

30

14

9

4

1

45

45

10

1

1

1277

1

3

120

1

36

5

5

1952117189

43

1377

11

111

7

15

152117182

16

1776

75

1

1

3242

176

86

1

3

6

10

183

183

7

7

176

21

153

2

2

199

1178162474

117816147

117816147

466624

461484

514

49

30

9

10

133

31460134
2771

712

41812

201

563

5

23027132

602

602

32

32

132

132

87

1

390

39

39

217

217

380129

339169

518

3578

14

14

14

14

21

21

21

21

21

286

22695

484693352622
1

60

265693241580

21

2341270

2341270

2341270

54

8342

8342

8342

18253

26

3

3

23

311450
6011464

29114
27114

2

139923552

139923552

6

1

80923515

1

50

1

72

28

7

61

18

2

29

316

10

110

106

6

10

105876
20

2

15

3767

316

1

1417

152

9

3

4

61

41

41

2

1

2

4

2

1536

7

102

4

92

7

7

85

85

70

5

10

5145

2514551

2514551

132867319534

1
53966410231

7

18466410131

255

255

184

71

661

661

661

26

7899933

63953

63953

3

7253

499

65

1509403

1509403

6840

8293

5

5

5

5

130

130

130

3

3

112

15

80

80

80

80

80

57282

304417292101427055852

6

6

6

6

233179

233179

1

1

213133

8

8

1

38

38

134111475

13211913

81181

8112

79

1112

1108

4

31108

3136

39

33

11612

14

590

118

2
2560

52
2555

150

2160

193

3

3

2

2

2

11552

7141

7141

7141

32

33

1136

17991718533220712

12

35911034141

120

112

8

4
23911034141

12

11182

7

2

222

1

2

1

1623

2

1

12

1

17

15621

26

19

1

1

1

32

3

1

1991

139

1

2

62

2

21

14065965328

622

2

2

42

5

71280

3751616

21116

1

11

616

6

1

319

377220

7

9

33

4333

1

187

14

1

133

476220

5

13118

18

3592

1

12

6

298148420215361

293147920210915
2

2

16

1

1

40

56126

1

49

84147817410915

43

451

3

51

1

14346

900423110

1

1

54192

3

11

47

392

4

3

31

31

15

15

438654

438654

3

103651

2

1

3

2

231

3264

1

391

12

12627

2

59

21

2

104

1

9

5

132

24

1

35

35

26

68

19

33

16114

16114

16114

3028

3028
5

11

1428

31

2

1

1

11

11

411

411

411

61

52

5

1

1

21

21

21

1263

1263

1263

511293

511293
17

51228

51227

1

148

151113

108715570477245688

5113

5113

1

31

1

11

1

1

6664

6664

6421

243

178459

172459

172418

41

6

6

10

611

2

2

2

28291061

28291061

28291061

12
511

391
111

28

742023511

11
742023511

8

86117

31

16411

12

61661141

195794234238858

155744153237347

433033026626

1

8

1

3

82555952

521

19

6129159200114

1

611

30564

1

1

3

1

2

347811511

2

2811211

1

431

12

1

13617531681315

6

6

37

37

1233

13

23

171

1

71

3

6

12

12

1

1

1

14452493

62

2

11

3

1

20

4417493

881298109312

1019

78129810833

1213

1213

1213

1

1

1

93

93

7

1

2

2

2118

2118

368

174

1

1153

1

1

1

1133

5

4

6

1

3

943

1

1

1

2

15

15

15

13

2

1125

17

17

17

1

1

117

1240

42

833

5

5

5

2532

1

1

2032

4

31

31

31

11

2

17

17

17

17

456

226

6

1

5

3

1

2

1

18

14

4

23

23

23

423246

4242

4240

17

1

30

1192

21

2

2

234

234

234

11

14

14

14

14

16

5871053503517495

673

613

10

1

9

63

60

60

50884950051736

2

2

1

1

521821167

26104555

6

55

219855

2678612

1678512

101

2

1221

1221
1211

1

1106

1

1

10

10

6

6

7

40518633461

40418971

7

400188

411

182

182

22257

22257

32

20

12

44753

44753
1

24751

1

2

11312

11312

2

6

1

125

15

15

12

3

431329210112

1

1

11362

7

2

58

1

1

1

3

3

3

3

2

2

71284

1013

6271

1

2
219136

11

126

1

1

1

166

28

1

1

2

14

1

3

2

1

11

11

169312

4112

21

1

13

63

24

1

1

1

1

31
191641

1611

1

16

1

63

4120433

3420
2

316

1

216

51

23

23

22

11

8

3

20413

5

5

1

1

1

1

12

12112

43234

1

33233

33233

3184

48

11

2

1

61

61

61

61

94

2

2

2

2

12822

12822

22

22

4

18

128

128
110

15

3

27764412359

260916323

4

4

1

3

8

20545821

419

2

19

2

1

1

1

457

457

571

1

561

172611

96

66

156411

1

1

1

1

259
1

258

3721052

5

5

5

14

1

1

13

13

141

2

1

1

139

139

7

7

5

1

1

4

16027816

25
45

2

2

226016

225816
226016

2

94

6013

743

7

7

7

9

323206

143

345

14

2

2

2

2

8078171371543

6524

2919

1

1

40521

3641368142

31413106

31413106

3

13413

7

4

4106

24023

23

23

11

1

1

39

38

1

21

21

1

11

12413

12413

11

11013

3

3

165357

164357
10

33

16

17

112

112

9357

1

4

1233992312

3

5019123

12

3819123

319123

1

15

19

76

5208

96

38

1

3

1

31

2

11

34

30927987

1386324582251

1386324582251

3863025118

1
3862725118

748204

3787924913

3

3

122531071

122531071
129

122529501

20

5

3

131171

131171

71

71

71

61170

1

10

10

10

10

5

1

4

1678

1678

1678

1678
1302

7

3

91

185

18185

18185

14141

14141

311

39

109

812

425
444

19

482213551532164

27812

16

27

10

17

10

4412
22512

7

2

7

56

6

93

10

5171219115135

1

113

113

113

72

137

34

34

13

1

3

183

28464

1164

1

64

10

273

31

36

1

47

156

1

1

412191449

412191449
321121

2

990

26

12

225

3

1061

102

102

41

1

4

138136373

893

4913637

171

11

2

4

10

3

2013636

2013636

7

7

2

2

267163

1491

1491

1461

3

11863

36221951

257161
36221951

19

33461790

33461790

5221143

1

1

1

1

1

2358069669119648120674837

21610360234549485954374

5

52

52

1

42

213

2791975166

24611165

3

331

1

91162

461

75

281961

11

17

10196

54

54

23

83303

83303

83303

37

2

2

2

1111

1111

111

1

1620015

21
1620015

611

81994

651

65

31

34

1

1

314212

321343

321343

21

2042

813

2

2

2

66

157515890123910481608

14

1

35152541723865
1

113

8937153

2

2

13

4661736

2

3

11

2131

11

11

766777

1585063733

61012

22337

37

223

2
21310414

1

13

61

1

110314

26726

211

21

1

1

51813280

2

1

12

3

21

1146

2

4111

166117

711

1272

38021

787

10

11

3

1

3

34

2114

5

113

1

11

31

3
91024298306

15

6

6311

1177

83

11824514

1

5

6

51

1

276629156

1

213

1

113

2

1

2670958385145
12418

10

1711

23962281

14

66241572

25013

594

965

21211

8

32

315

2164063

121

531

2

112624158

137

1

17

32213

1076

18

66

31

26571

122

11402244

5

343416

82

21

349

1889

1

16

31

26

1

25

3992

26

1292

1

8

2

2

1

3

2
886124211267586

165

31210383

8085

464102411

1

11824

1111811002

183182132

1812

1

1

6

133

49151

7020

1591

1591

41

7

1111

601103

601103

51

551102

81

5

5

5

4041

4041

3781

18

8

10445125

10445125
1

607

55644

120121

22

4131

3

3

3

3

781287724224

6812870220

54

201

1

3315

6094

64381

1

231

1419

323

3

1072224

1

1

2

1

23

622222

1

1133

211

391532

391532
10

11

1

2311

1

31

142

1

146

146

23

113

10

3

2

431935

91295
6

1068

3

4

212

11

1640

13

637

32

1

1

2

2

7

7

6

4

2

1

1

2575300743408745117

22

3

10

8

1

1871
6

94

1

231

2049
9

8

248

1

1

3

5

2

3

1

1

12

12

1

1

4

1

1

2290

2290

1212

1212

19

3

7

2

7

183
126123

1

1883

2

52

819

2

9

7

20109

19109

1

19

19

5

2

3

4941229

1

8

1

1

4831229

33757154966412

27

1

2

30757154696412

46

2
16122482764473572

5333

20

8

52094838

1010

95271

251032748773412

2

3

20164

14

329

329

2

2

18193

18193

17193

1

4445

122

1

17

1

53

25

8

1

16

3225

92

193

294

723

723

7

23

43

84

13

1

1

2

21
1

2

5

5

13151

2

282114315

282114315

12

51

41

4

2

195

2

16163216

61

18132212317

16

16

1813221231

1322123

2

161

91

21
4223269410713

84

84

442

442

30312

1

1

39

29911

3

3

111211

3

2

1

16411

36

6

137385412
6

550

3741

3

11

641

1

81

554

2

74424311

23

1361

7

7

59233

24

1

12

321

31

1

1

13781

137

81

2

2

3

3

5351

3741

8

153

18

18

57

57

27416381
2

1

1

22

237

964

1574

2

1

61053

1

51053

2

2

1844

4

1804

42

2

3

1

3

10

7

52881

52881
30

378

721

474

1

4

3
18

2

2

13

5

1

7

9

1

1

1

6

4

4

4

4

181

181

181

181

194833646741543472462

24524405423591110178

1

1

14

6
389720523425

11

2

21

18

758

59

4

13

1

591

6202

31

8141611346

118712

1

31

90

67

23

14

1

4

194593

154553

44

19

18851

121

1

14

580

7142051

26

41

49

1

1

13635

1

8

8

12548

12547

1

16563332891144
1

116

17

11

6

78

3243441

1

2

528623883

16

12124533242

1

6322992641616059
1

1972079128

18

152

10

19311512

42

51

32

1

172

2

11

1

15

211

218

1

11

16217273461157

4

337

11

1258829

18749

135213

420

14

46

144

144

14

13

3232918

49

49
1

39

1004339291501901135

118
1004339291501901135

211

352652112184105

6193227811416

11

1172

153630217261369

33868411

4613

1223

1117119975

12

1463067

221

25982534

121

314

82923

1

673212

173

99

99

74

227191160

23
596

34

1

29

2

2

1

7260

1

3

11

3258

1113

3

3

1919

1

1419

4

28

14

2

12

640

640

94

544

2

19

19

19

3332311

22269

22269

11632

3555583151

21

4
4611

33

911

652

1

1

3

31

84

1

83

17

1

133454315

311

211

1

1

4419

4419

1

1

3918

4

522123222212

522123222212
24

2

3

1

1

11

1

5

2

3

65

5835

34

7095

41

4

1

3

1

1

1

76

2

1

2

131

1

2

6

1

1

3

1

1

153123632

6

2

13

1

2

5856

2

7535851214938

1035841213938

2838786

111312

673211111

31927

24

16

16

4911

3

1

1

6

8

3

29

71118

1
316

16

2

458

11

1277

1277

1277

28

28

28

1105

1105

1105

2624

2624

13

6

2010

1

7
224366

71

84365
31

1

2

4354

3

6

6

6

159

141

141

1

1

17

5470451

22
5470451

50

1

121

1

1

469

6617

52

2

5

6115

1

515

1

42101

42101

4

2101

4329107180351028

333224

1493

13

13

1

81

21

1

59

1

1

58

3

55

7

73

6226

4915

4915

51

4414

695

695

695

59

3

3

45

3591108

3591108

1

1

1

1

1

23

23

32

32

3221105

10

34

71

271105

22325

22325

22325

22325

172291

181201

181201

181201

181201

273

273

16

113
4

1

2

3

4

111

111

1

1

11

183

183

183

183

68

85

85

85

75

1

311803445

311803445

311803445

11894

16136

345

4

281621

281621

476
1

306

13

156

96

1554

926

628

69021

6

1

13221

3

435

113

58716

753

753

753

51213

46

69
1

32

5

8

8

15

4016

1

94

53

831

14

117

9

17

981

8

18

16

2

101

1

21

7

2

2

1

1

1

1

1

2

9323420

4

4

3270375

145

145

1

53

53

3701

12173

12173

9973
12173

8

3

11

527515271716

119216

119216

9924

9924

16

16

412

412

122198

286515251502

84283241

7

928324

3

1

1

328324

1

27

27

381

211

17

3

36

818129

2311118

2051280

2051280

2051280

1

392

133

43

4932

61

61

5

11

332342195428612

23225423990

1054730

1054730

1054730

222223260

33

33

33

33

25114

455

67342174450

67342174450

67342174450

67342174450

1

5512120

5512120

5512120

5512120

1

4

35

1211

2

11

3

18

3813
2

61

61

61

4

21

3012

9599

9599

9599

9599

85185

215120

4

867

867

867

615

10

3

3

3

3

3

12

91560

91560

91560

49160

44524

4636

424

107

9

308

50111626

2811

1121

11911739

15

11

13

351117

2

2

2

5

5

231117

5

3522

5700

5700

5700

5700

451

14152

734
544

19

19

19

332

152

1

28111279544018

28111279544018

287972

37972

37972

25

25

1112536818

1112536818

1112536818

30
11424055342964

9

2

111716

61514

52

52

4

12

2

2

2

22

4

1021361

8111

8111

7111

1

1

1

1

11135

1321282

1321282

30
32122132

2113

2113

2113

119322

1

1

1

1

2

11

1

2

5

33

1

21612

21612

21612

21

710

91

161227

112

94

623

21

2
62

42

152141261426

152141261426

152141261426

91

2141414

3

312611

1

252

21

39294

48981410308349618

4631681637

6661596

39

2427

2

19816213

107

111

16

7

733832222347594
583243

43

6

51

1

151134

166

11

54

10

24

5561

223121513365

262715

262715

262715

4613

6

15927

3

503

35

574

35

2847

10

122

3422533015

3

21
1143186385

13

24

3

2

8

1

29

11

213186361

5

6

3558379
1011

103

2080

21

161379

182

132

132
102

2

1

1

781653703466308213359

861

751

21

11

1

1

1

1

73

73

73

73

73

73
16

54

3

11

11

11

6

6

5

5
