# Supplementary material for: Revealing the Microbiome of Four Different Thermal Springs in Turkey with Environmental DNA Metabarcoding
Source: Biology (Basel). 2022 Jun 30;11(7):998. doi: 10.3390/biology11070998 (PMC9311576; doi:10.3390/biology11070998)
Supplement: Supplementary file 1 [file biology-11-00998-s001.zip › Supplementary Data S3/515-806_Forward c2l100 krona/515f-c2-l100---ssu---krona----Total---sim_93---tax_silva---td_20.html]

Javascript must be enabled to view this page.

magnitude
magnitudeUnassigned

515f-c2-l100---ssu---krona---515df.c2.l100----Total---sim\_93---tax\_silva---td\_20
515f-c2-l100---ssu---krona---515kf.c2.l100----Total---sim\_93---tax\_silva---td\_20
515f-c2-l100---ssu---krona---515nf.c2.l100----Total---sim\_93---tax\_silva---td\_20
515f-c2-l100---ssu---krona---515ngf.c2.l100----Total---sim\_93---tax\_silva---td\_20
515f-c2-l100---ssu---krona---515yf.c2.l100----Total---sim\_93---tax\_silva---td\_20

15842135967806979011318

3911225225155

1

1

1

1

1

1

1

1

1

1512712381779297605671

111

111

111
81

1

2

4074316276

61351

51

1135

1135

1135

261

11

3

1

1

2

1
22

5

1

1

4

6

4

194

8

8

8

114

323259

323259
25

294259

294259

4

33301111

6

6

1

1

5

5

206

20

12

1

1

1

4

1

6

6

13025

13025
15

22

1

8

1

1

3

3

3

5

5

5

212363382

212363382

278

2

2

78

78

1236262

1236262

1236262

11

11

11

3
11

1

1

6

49

1

44145

20

20

20

20

1

1

1

1

4

1

1

3410

3410

3410

2

6

1

2

41

49

4

11

3115

3115

3115

5

1

310

2

22

3803054256301

69

6

6

6

9

9

9

346229425413

2622813

9

122812

12

160

1

44

6

17

1

161

11

13

2

40416

4

1

11

6

299
25

49

1

3

14

14

1912

6

4

2

61

6

1

3

3

18

25

18

6

1

241

241

5

1

1

1

18

11

1

2

3

1

1

5

5

63

17

46

12

3

3

72262

5226

5

226

2

2

2

1

1

6636

493

93

3

2

2

1

16

16

4

1

1

2

2

5
96

8

1

1

1

1

16

542260

542260

6225

5219

4

4

120

566

566

29

16

253

253

25

25

3

17

17

15

2

13

2

2

68

64

4164261
3

4

16141

16141

16141

16141

2

2

2

1415

1310

1310

1310

14

1

95

95

1

1

1

1

7

7

7

7

7

2813304

1

26839

11

11

11

1

1

1

3

3

3

3316

22023

198

6

189

3

2

2

18

18

4

4

3

3

13213

41

9

183

183

2

2

2

1

2232

6

6

4

4

1628

19

32358512657

2352095

222091

154

154

154

410

8

53581544

53581544

53581544

53581544

389166186

34

34

34

34

114

114

114

114

7

7

7

2

5

6

233

233

45

20

25

62
2

8

4

6

39

3

117

45

72

9

1

1

1

1

117

334

8

8

12

12

2

1

2

16

11

10

1

5

5

1661

1661

1661

1507

154

2

2

2

2

417

417

6

6

357

1

297

5

677

532

2

2

2

2

2

381

1

1

14

11

5

5

2

11

8

3

2

1

2

145

145

145

30

101272

101272

101212

17

17

27

27

6

6

1
12

8

2

1

59

3740

3740

43

43

17

31211
136

12

73

28381

51

11

30791

457

26061

16

6

6
2

4

6

51019

18

16

16

2

2

2

3

3

2

2

2

2

1

1

1

1

5

2

1

1

1

1

1

246156821481428

2

38

38

37

1

1

80

3

3

1

1

1

1

10

10

10

10

1120

23

5
23

18

197

6

6

129

17

22

62

1

60

1

42

522

522

522

522

52

2

2

2

32

3

2

1

2

2

6814461963315

35431961510

52

52

3543196108

276

201

145418910

1671

1

1

4

10541

10541

10541

1

1

1

6862

6862

678

82

115

115

3

112

61

61

1

1

1

1

3

22

22

22

15

15

12

3

2
7

2
5

3

11140122

1

1

21

21

2

2

303

303

711072

711071

1

27

7

2

2

2

167122134828

2941

2
2141

5

1

13

21

2

71

1

8

8

10523

453

3

1

1

1

15

2

1

1

6

1

1

2

2

2

1

1

1

1

381228222

381228222

5

3

111228192

4

1

2

53

4

3

20

20

20

83202

1

1

4

4

5

57

17

4

1

7

1

2

2

2

2

111

3

1

1

3

3

1

437

437

44

2

1

1

4

2

2

2

1

1

88

88

88

82

6

36

4

2327630510679269421

2042573272598240

59131

59131

1

1

2

5

6

11

2

12

1

6

2

15

2

5

6

2

2

2

5

3

34180083361618

1

2
13168285804

2

2

1

8282

1

4228

3

1

116402694

1

11

9

1

1

17

16

1

1

1

161

15

1

1

5

5

17031876

3

18

2

701621

5

1

5

35722833

322047

5

570781

2

2433

12

1431

33

33

21

1

2

3

3

3

22

6

16

16

1039617721828

1039617721828

13304712

5

14

23711461688

746

110

12

22

1

2

4

4

4

4

3

8

211

1

1

111

111

25

25

25

11

11

1

1

73315802

6

1

1

4

13315802

51

128573

4671

3

3

3

1

1

1

7

7

7

3

3

3

1

41

41

41

1

1

111

7

7

7

285

5

5

7
1

4

1

1

2

1

1

5

2

3

14

54

54
50

4

1341

1341

341

1

2

2

2

212337327953287381

1

36

1
36

5

1

2

3

3

2

1

6

6

3

3

2

15932669330941

40

40

21

1

18

2

2

2

152526871986

1

22521

36861979

2

11

93

1

1

192

192

6

6

371

131

24

574511011

574511011

5

1

4

25

5

2

2

2

2

2

1

1

8

2142

2142

12

114

183177110293176

113

1

12

1

10

10

46

3

43

13314838251

131

131738

8

242

1

14014

11

1

1

4

4

3459211114
5

1

81

4107

41

1

2

145922

476850711

1

431

1

2

4

11

30

1

4

1

27029

1

458

1

2402

1

2

2

1

1

190

1

90

9

216837

16736

1

2

1

3

1

2

1

12

12

2

10

1

1

1

215

215

215

16

101810

101810

31

608

1

574

16

365

1

1

1

2

454249352
3

359

357

2

21

21

7

2

5

8852

2

5532

3

30

29

29

1

1

22161

2

161

19

1

45

15

29

1

3

3

5

5

4

4

2

2

1

1

37

30

1

2

27

7

1

1

1

4

8123271

17

17

712321

1

3

585

617

2

301

1

1

3

3

1

2

2

181

2

2

2

41

1

1

1

1

1

45

45

17

28

3183

2

2

53

53

130

115

15

1

1

4

11

11

8

1

2

3

3

2

1

38

38

38

1

1

1

100

1
98

8

1

82

6

2

1

1

41346

41346

2

21346

49

3
49

4

6

36

1

38371127
6

25371100

1

337

337

337

432
332

1

1

3

3

14

316

11146

11146

8143

3

3

12

1

2

10

10

10

6

1

2

1

218

1

11

17

1

3

15

698

698

698

378

43

335

32

10

21

1

10647164

314773

184772

1

10

10
4

2

4

3

3

3

7591

687

687

397

17

12

721

721

22

51

18

18

18

18
3

14

1

2

2

2

2

2

398

13

6

6

6

6

1

5

45

45

45

45

3451587112

28028
69

1128

22

7

171

3771386
3

3

13

4

9

42

7

3

129

78

1

1

1

1

1

10

1

2014

2

2

1

1114

2

2

1
845426

145426

2

3

1

514

1

1

214

46323162

1611

20323150

1

1

2121

154

23

8

1

1

1

763

2

1

1

163

3

1

6

1

3

3

133

3

219

219

219

101

101

81

81

2

2

19

19

17

17

2

15

2

2

795715132

1725

1725
1

4
1

2

1

11

11

125

236810

22

2

2

2

2

51

51

21

3

2367

2367

136

7

1

1

32

8

30395

311

162021

2

7112

1

6112

1

4

1112

11

19

1

4

1

3

13

91

91

91

3

15

15

15

3

12

14

11228

11228

2

3116

112

12

1

12

12

12

12

12

1

31684163

31684163

3168601

1986600
3168601

11821

3562

3562

5

2

3555

3254313522

1

9

1

1

1

15

1

1

5

5

161

161

313831354

3

1

1

1

513111

35

1

25

28111

11

8

111

10

10

10

10

10

221198

5

5

2

2

134

134

22112

1

2212

11

1

1

1

1

13

3

1

1

418122

15

5

1

3112

1

1

11

12

28

2

6

2

13

13

11

11

11

323109398492

45

45

1

5

5

5

3

3290

3290

3290
3289

1

3

3

3

3

24

1

23

23

4710457341

212

3747

2

2

2

374

374

374

5

5

5

26671339

65930

65930

65430

5

26439

26439
51

9

10

2438

8

8

8

3

3

3

3

3

1

1

1

1

1

7

7

7

7

7

23348156

23348156

195
23348156

12

348155

10

1

121

121

22

1

1

1

1

1

1

1

1

2

2

2

2
