# Supplementary material for: Revealing the Microbiome of Four Different Thermal Springs in Turkey with Environmental DNA Metabarcoding
Source: Biology (Basel). 2022 Jun 30;11(7):998. doi: 10.3390/biology11070998 (PMC9311576; doi:10.3390/biology11070998)
Supplement: Supplementary file 1 [file biology-11-00998-s001.zip › Supplementary Data S3/515-806_Forward c2l100clean krona/515f-c2-l100-clean---ssu---krona----Total---sim_93---tax_silva---td_20.html]

Javascript must be enabled to view this page.

magnitude
magnitudeUnassigned

515f-c2-l100-clean---ssu---krona---515df.c2.l100.clean----Total---sim\_93---tax\_silva---td\_20
515f-c2-l100-clean---ssu---krona---515kf.c2.l100.clean----Total---sim\_93---tax\_silva---td\_20
515f-c2-l100-clean---ssu---krona---515nf.c2.l100.clean----Total---sim\_93---tax\_silva---td\_20
515f-c2-l100-clean---ssu---krona---515ngf.c2.l100.clean----Total---sim\_93---tax\_silva---td\_20
515f-c2-l100-clean---ssu---krona---515yf.c2.l100.clean----Total---sim\_93---tax\_silva---td\_20

5184158975819652747

3231099851

45

45

5

5

5

3

1

22

1

1

1

1

2

2

2

2

1

1

1

1

1

1

320

320

319
320

1

3

3

3

3

471047321

212

2674319

26439

26439
51

10

9

2438

6210

6210

5

5710

8

8

8

307

5

5

5

30

30

30

2

2

2

3

3

3

3

3

1

1

1

7

7

7

7

7

24

1

23

23

23355

23355

23355
195

10

121

121

1

354

12

1

1

1

1

1

1

1

1

1

4501145274419351371

4621345

2021339

1

1

1

53

214

215

215

215

3

763

163

3

6

1

1

3

3

2

1

1

154

23

5

1

1

165

101

101

81

81

2

2

7

7

7

7

7

833244

7039

1

1

1

3623

3

3

2

2

6

6

26

17

6

3

4

4

3

3

3

2616

4

4

4

1

13153

2

2

2

9

123

123

41

91

91

91

71019

13

33

10614164

311473

181472

3

3

3

10

10
4

4

2

1

7591

687

687

12

17

397

721

721

51

22

8

8

8

3
8

3

1

1

210

13

8686781731642145

666365118475106

215

215

215

1

1458

1458

55

32

10

165

1

61

24

24
3

11

6

4

111

29

29

22

7

10526503261

12

12

5951301

5951301

121

61

6

2

2

25

5

2

2

2

1

1

20

2

1

17

2

6

6

151744189

2

93

2171

343182

1

11

192

192

2

1

1

5

1

4

2

38

38

38

1

1

1

4164

4164

2

2164

1

3

3

1

2

15164342
3

4

4

5

5

16

6

9

1

21

21

1

1

7

2

5

28

28

87

1

86

3052

2

21

3

632

2

2

1

1

3

3

2034

34

17

1

2

3

3

1

2

77167686160

113

1

12

1

9

2

2

18

8

1

312429229

81

1912

1

242

5

29522

5

1

1

22737

1

2636

2

1

46

3

43

3

1

2

4

4

30401120
4

2

101

214

41

1

1

10402

8

8

1

1

1

46327391

1

4

7

2

292

1

3310

1

418

11

1

1

1

1

4

21

272

272

17

12

325

8

8

1

1

2

2

17

14

3

6

6

3

3

1

1

1

1

1

1

9

9

2

1

6

8

87971

17

17

7791

101

37

2

1

32

1

3

12

12

2

10

1

1

1

1

7

7

6

1

4

41

1

15

33

31
1

6

8

15

1

2

1

1

2

2

2

22

15

12

1

2

7

1

4

1

1

2

2

36

1
36

1

5

2

20231355116739

210

110

110

1

1

7

7

7

22

16

16

6

3

3

3

11

11

1

1

265

2

1

1

7
1

1

4

1

12

5

5

5

2

3

1

1

33

33

1

2

21

59131

59131

3

3

2

2

2

6

2

6

6

5

2

4

2

1

1

2

12

1

11

3

15

41

41

41

341912682618

17

1

16

1

161

1

15

1

2173

1171

12

11

1

9

1

1233616

1

2

5

18

3

5

23361

1

1

2
1314651024

1

2

1

1

1

818

2

1138454

3

538

5

5

2

3518647

4

32546

51697

23

23

23

1

1

1

22

4

4

2

3

4

4

1

8

3

3

3

1

1060172537

1060172537

55432

6

745

5

24911190

14

17

15

15
11

4

2

2

2

7

7

7

111

7425252

1425252

51

2171

12118

6

1

1

4

3

3

3

191

191

91

1

43464
3

1415

1310

1310

1310

14

1

1

1

1

23

23

2

4

2

2

316

316

316

316

1

315

266238

266238

197

197

5

190

2

26641

16540
26641

1011

44134

13

13

13

13

1

3111

3111

3111

5

36

1

3410

3410

3410

4

41

2

11

6

1

2

49

4

1

1

1

1

1

18

2

79271542

3037

217810

51

51

3

21

22

2

2

2

2

1

2177

2177

117

1

7

1723

1723
1

123

11

11

1
4

2

1

16921

4

1

3

1

2

732

632

4

132

1

1

11

16

32

8

311

6

286082

286082

8532

8532

8532

278

2

2

78

78

49

19

406191633

6451

145

145

145

51

261

1
22

4

5

1

4

6

1

11

3

2

1

1

194

114

8

8

8

32618

25
32618

29718

29718

4

2915119

166

16

1

1

8

1

4

1

6

6

11525

11525
15

1

8

7

1

3

3

3

4

5

5

5

6

5

5

1

1

1

5

2

2

2

2

18

2

1

1

1

1

16

16

2

2

2

2

3

3

2

2

54

1

28747632

8739
1

2

3

1739

1

3722323
3

9

7

3

3

110

12

13

1

4

1

1

1

1

10

15

1

209

2

119

2

1

2

2

2229
37

145

22

119

7

14

19

29518261

677

532

11

2

2

2

2

8

2

1

3

2

381

2

14

11

5

1

5

1

2

145

145

145

139

139

31

20

11

2
43

8

3

6

20

4

9

56

38

18

112

113

113

113

113

16

16

16

16

182

182

182

166

16

7

7

7

5

2

2

2

2

2

416

416

6

6

356

5

296

1

6

334

12

12

2

1

2

8

8

16

11

1

10

5

5

1

1

1

1

16

16

2

2

14

14

2

12

322551286

8

525142

525142

525142

525142

48

235228

154

154

154

22224

1

1

13

13

13

10

3

2

1

1

1

1

1

3816194
6

1

27

6

1

11

2516178

3

3

11137

11137

8134

3

3

312

1

316

316

316

14

1

323
423

1

12

1

2

10

10

10

1

6

1

2

111

111

111
81

1

2

2

1413

4

4

6

6

89

3112

1

12

112

1

12

8

8

8

8

4

468

468

468

158

115

43

31

20

1

10

2

22

6

6

6

6

5

1

3

1

3

1160

1160

3

14322

14322

4

4

4

14282

17

36

36

457

457

28

3991

48

15

3361

3

3

12

12

15

15

12
1

1

8

2

4491
23

30

3521

12

11

21

7

3185947251

73

73

7

7

3

52926

52926

17

17

2

2

15

13

2

634

16

528

3

3

59

59

9

7

66

6

6

6

6

6

6

284174708

26498

9

161

13

2

11

1497

1

13

6

7

1

9

20

1402

18

5

5

6

2

4

23

1

6

16

3

3

1

12

18

11

3

1

1

2

1

1

5

211

211

51

1

5

3

3

18

11

7

1

7142

514

14

5

2

1

1

2

2

16

6435

1

1

2

2

1

1

4

4
95

1

8

1

473

93

2

16

1

1

3

14

1

31412

16
205

45

4

1

6

3

11

14

14

1

18

18

18

18
3

1

14

1

2301784477222

3

3

52151293015

112292

1

1

83

83

2

2

21

21

71442

1

71441

1

1

1

402

402

72

33

9

9

6

3

791

791

791

372291510

52

52

37229108

1111

1

61

1522710

326

1

2
7

5
2

3

4

61

61

1

1

1

1

3

45

45

3

42

7

7

7

11

10

10

10

10

38

38

37

1

1

1

1

1

1

191

22

22
5

17

169

6

6

39

37

1

1

124

17

17

2

16

16

16

6

10

514

514

514

514

16727104725

1

10523

453

1

3

1

1

15

6

2

2

1

1

2

1

1

2941

2141
2

1

1

13

5

71

21

2

8

8

2

2

2

1

1

1

17

17

17

1

1

2

38275212

38275212

4

2

53

5

3

11275182

4

3

1

83202

2

2

437

437

7

1

111

1

1

3

1

3

3

1

1

57

17

4

2

2

4

4

1

5

44

4

1

2

1

17

27

7

2

2

2

52

32

3

2

1

2

2

2

2

2

1738243222

1

15

1

1

5

5

163024324

11

1

1

1

1

11

11

11

41892

13

13

25

2

2

3

15

5

1

3112

12

11

1

1

1

1

1

13

3

1

1

3

513111

35

25

1

28111

11

111

8

2

2

2

71128

2

2

84

84

3

3

7112

1

712

10

10

81

81

1

9

1

1

17

17

17

17

359285221325
