# Supplementary material for: Revealing the Microbiome of Four Different Thermal Springs in Turkey with Environmental DNA Metabarcoding
Source: Biology (Basel). 2022 Jun 30;11(7):998. doi: 10.3390/biology11070998 (PMC9311576; doi:10.3390/biology11070998)
Supplement: Supplementary file 1 [file biology-11-00998-s001.zip › Supplementary Data S3/515-806_Forward uniq krona/515f-uniq---ssu---krona----Total---sim_93---tax_silva---td_20.html]

Javascript must be enabled to view this page.

magnitude
magnitudeUnassigned

515f-uniq---ssu---krona---515df.uniq----Total---sim\_93---tax\_silva---td\_20
515f-uniq---ssu---krona---515kf.uniq----Total---sim\_93---tax\_silva---td\_20
515f-uniq---ssu---krona---515nf.uniq----Total---sim\_93---tax\_silva---td\_20
515f-uniq---ssu---krona---515ngf.uniq----Total---sim\_93---tax\_silva---td\_20
515f-uniq---ssu---krona---515yf.uniq----Total---sim\_93---tax\_silva---td\_20

1185301023846555184151117590

293193151331764150

251843

251843

201837
251843

56

2322

1

1

1

1

122

122

20

20

12

1

2

21

21

21

927

927

927

927

927

12

12

12

12

1430

1430

1430

269

263

263
258

5

6

971441

971441

3

7

144

144

144

61

1

1

1

26

1913

113

113

113

1

12

1

1

1

5

5

4
1

3

1

1

12

12

8

8

4

4

264

264

264

5199156120321706

30713

7

7

7

7

2126688321686

191101321182

387
191101321182

42

39101321175

72

1

36461502

36461502

36364502

97

31

41

4126

31
4126

1125

2

2

4

4

2468120

41

41

41

2468

2468

2468

79

79

79

52

2

1

4

1830159535

1830159535

1830159535
1455

776

746

3

17

94

131

56159529

10963490696648138295043673
1

248224744617749

438

36224742591

36224742591

36224742591

36224742591

3084

17844215036

84428

84428

84428

170215008

20781042515756803633

2152

2152

1

2045

7

7

7639446142732968

143940142316647

480377679

31

64

1

11

1

21122

2

373440609

42

34

30133

10313613469838
2

1757115

3

3

4

5151311818416

1

1650162114

21083

2

1

1

1

1

41

41

31

1

1

1

1

8

123337

6

6

117337

2

24

117311

85

85

1

5

4

3

49

882

2

1

1

1
862

1

15

50

2

7

7

1

4

2

2

2

1554991

1554991

1554991

365618

365618

35691

878

1161157411811

1011

1

1

81

1

3

3

12

12

668783798

558783793

115

192

192

17279343

4

6

6279343

1

35
6

29
5

24

2189

2189

2189

160

160

133

27

489

567

5

5

5

66

1

1

1

285

165

1

1

1

14

11

3

5

5

12

12

12

15

14

1

1

4

4

4

4

61442

61133

1286

217

169

563

62

5

1

784

3

689

92

309

309
46

1

262

11

26167

26167

26167

26167

1

2163

2163

1163

1163

1

162

128

128

1247979120351236

6491604

2

903

15

21

7

40

6

1

2

152

2

81

13

13

51

4

4

40

3743

6

1

4

6

19

242

4367

3

39

167

12

25

25

6

6

2

32046

32046

17

4

5

5

5

7

7

7

78471821

254721

6

23

5

6471

16

31

2

1

2

2

5

327

3

2

11

73

53

41

169

5

1

1

24

23

51

1

91

91

2

71

10

1

1

1

247979721498

1
247979721498

1

3

63978721248

15

5

23

41

27

16

13

2

4424

30

1

1194

1194

1194

217

117

1

218222

76

75

1

142222
12

1

816

3

2

1

7

115

562

12

24

31

2

2742

2742

2742

235

707

31

31

31

31

8

101

101

101

10
101

82

8

1

1

1

1

1

351

351

341

191
341

3

1

8

3

1

1

1

125

125

125

3

3

1

98

23

4

4

4

4

686101138

686101138

24

24
6

4

10

4

685861138

197

259136

258966

17

126

126

741
8

131

44

9

807

807

196

196

14

14

20446125
561

1

436

653

74

1023

18619122

358

358

2023016

193

2508

1752916

225

46
419286131273

2362861950

3

5

1061362

3
1061362

2

1

1

631329

23

2

1

21

391

3

19286

19286

19286

9226

36302
58302

20
22

2

19

542

31

28

3

3

23

23

23

1
98261

54

2

5

6

2

12

4

110

1

10

10

3240

131

3

15

56

3

12

6

68

58

58

7

7

40

4

10

10

83

1

3

6

53

5

3

16

1

13

13

5

8

8

8

26120

281233887342

281233887342

2805636682

1685333662
2805636682

1279

11076293

673520522

1
673520522

3514922

6744

11

6

1

3

2

63160262145
20

1

9910

3710

2

42

42

1

2

3

1421077

1421077

4

2

43

41

1

4
3

1

2

14

21

61

6

6

6

1

41585
1

3

2

11150

11150

11150

1

16

2

2

2

215

9160924

9160924

9160924

5

160924

4

17

2224364

2224364

1005

1005

1005
755

6

11

3

5

667

667

667

8

8

8

46197

10161

10161

6120

6120

1

37

4

2109

441
49

32

83

83

83

83

83

3414841329016751

45

13290

13290

13290

951

12339

26244

26244

26244

26244

338169

338169

308169
2

12

26

14

249169

5

6

6

1

1

1

1

1

21

21

16149

16149

16149

16149

181

181

121

1

5

61

6

17115182

2050

2050

514

204

309

1

583
13

2

24

113

34

43

1

2

351

888

534

353

1

65

4

4

4

4

26182156

462

111

1461

501
1

50

2

2

1

8

8

85

3

82

188

188
1

9

28

2

4

518234

89

89

89

48

31

1

525

525

525

415

9

1

1

1

1

1

1

57836

11129

11129

11129

4677
22

3
64

3

1

2

7

22

5

2

19

16

5

11

121

11

21

12

9

3414
1

103

19

10

15

1

1

84

38

134

10

1

10127

9

2

2

2

2

6068128

19381234930

12822713

221613

221613
221513

1

44
2

24

24

88

347

180612122

7

7

7

3

3

3

4

3

3

1

5

5

5

268186

13961136

435

435

190

190

11581

48

62

10481

401

401

310

2

10

1

1

1

123

123

1

6

115

1

417

37009611221491

229151

1091

1091

3

1061

12051

3598759398

861

11

11

61

61

69

69

128

13

1

3

25

25

6

20757

190

2

9

1

28

133

3

4

10

1757

1057

1

5

1

57

57

57

2875839

2875839
1638

144

2

11

1

730

58

20919

1

1

1

7

13

4

4

9

1719

26
171

49

2

3

24

8

20

7

32

278711321

278711321
2031

255811320

255811320

26

11686272

852

318627

1
91

2

41

2

3

4

5
2

3

108626

3

2

1

48626

336519152920002276

157

157

5

5

152

133

12

7

30471113291985164

6053487

21

14

5

2

11

11

4

406343

13

3

9

7

1

148

2

2

4

2

5

121

5

7

71

69342

1

23

11882
77

16

87

3

5

7

5

222

1

1

131

2

2

1

2

1

1729303

20

1907

547

134

2

825

3

25

77

771

1

23

23

2

6

6

127

13

13

11

120

76

14

11

3

15

1

5

2

62

62

675

426

249

12

1

11

42

30

1

8

1

2

1

1

31316

31316

3

3

1601716160

1107

12

88

8

27

36

141716153

1

2

1

4

3

1

2179

492

981

1153

52

11

137

335291411

6

251
27285

2185

3

1

151

121

17

541

8

1

929

429

5

67107911

3

3

311079

7

1

3

31079

17

145

1

4

14

8

2

6

72

5

2

1

1

3

21

11

1

2

2

4

10

44124

44

1
44

43

124

124

124

423427

423427

427

234

225

9

631583

563

581520

11

3

261

808

49408

2

47408

542

542

5044415502

5044415502

21

177

91942016920

30941910419

16741910317

79

17
79

11

51

3

591

591

591

12

6101651

1291321

1291321

7011

5932

48133

7
48133

1324

27028

1

72

20104

445771

58660490615

1

15122

103264217

4

10

67

3613421

2713421

313421

13

11

9

4130

34

1

26

1

5

1

9

101

4

119224

119224
9

9224

5
29

16

8

72

72

3233945106

1179

1179

7

3

179

11

11

1

1

12320

120

1

20

23

1

22

3

29339177

29339177

1

11339

3

7177

7

22112257

1

47

1

32118

4

2075235974812

2075235974812

752355712

752355712

752355712

204048

34048

34048

17

17

3874

548

1

1

1

1

1

106

17253412106884786913168

15681227284872257102897

826

2793

2793

67

2186

45

1

28793871

11

11

28793761

111

2

1079373

3

1

4

1

5

403

403

137

261

1

1

3

39426

39426

39426

15

5

2311

2

45

1131

52

52

611

1

2

21

2

135

9

9

1

1

7

15

15

3

3

9

663094

663094

2

2472

83

62786

51

34364

80

2

271

38542

38542
11

9

211

1

1

2

5

1

132

491375

34

1

1

6

8

1

3

11

3

9

9

449

449

2
6926

2163

2763

55

55

30

25

2

11

11

1

1

24108

1
24108

16108

7

3704

22
3704

266

1

1

46

1

334

1536191740872404512

1

1

2647532805111

2647532805111

225

219

6

3

10

10

1
6

3

2

20

20

1363

1363

1

1

321

321

1

1

6

2

1

1

2
1625

6

1

2

1

5

24

40

8

6

8

1

17

346192

1

217

128192

173

1

1

2

157

10

2

14114424036159621

4

1

1

1214153

9018

122

45

24

2141

2814032158941

221

221

1

4

35

1

34

34

34

34

363

75

2

1

1

6

12

9

6

38

288

268

6

14

4657

4381
5

341

1

6

332

60

236

36

1

19

4

4

6

13

13

13

2

4

7

119

119

1

13

9

96

57

57

35

15

1

6

1

12

4421

121492

3

3

3

16

8

5

3

8

8

1

5473521855

45734845
3

42

3603

211

19

3471

2643

3

63

112

31

94181

1

1

54180

1

2

1

12
3612207722379

27641
9

1

9

2745

1

1

1

43

43

42

42

31

1

21

31

1

30

2

1

1

5

5

51

1

1

13

36

11

11

4
452

306

7

135

2
2021258

1581

17

4

3

1254

11

73

311

1

1

21

6

2

4

11817609

1

7431539

1

1

3331

1

67

46

22

2

2

14

14

62169

42169

2

3

6

1420104207828211341

31

64

3547

1

530

2

1

16

9288

121

187

5

2

14620

14212

3

8

1

30

30

175

31

1

94

4

18

2

16

2

2

9969883078468
2

975563323

5723

1926114

5

1044

131

202462

11

30232651215808
54

502741

6

133

291915

1

3

11

3

61

169323213

1

7

1

1

1110

610811

2

5

47

91

101

3

2

23105711268

71

2

131

3

2105311238

1

81

1

1

3

4

2444043646925

2

151

242

1

19

15

61

24217

2

116784287

5

533

4

22961

1122

60

2

126

131

1

17

28

831919881

1

1

15229

1

1

161479

161479

91471

78

44322

4332

4032

3

12

1

2

5

5

5

5

4

4

4

4

156318482201252981271

156

65

91

91

2520

2520

6

168

1

311

7

34

3

3

4

4

21971

2

5131

381

81

30

583

2

1

1

15

4

49

49

3

96259111671596464

96259111671596464
65

15233194613040

116

3

7186

22

3

5

6533

2

2

109216271112

4339

623550

157

3

402141606714

301

2

1

16

2

8

2

1

1

92140606714

21699588

3439741

46023

3

24

11

11

11

32593

1

22593

2

2593

13

13

13

1691134

4

4

1

24

12

12

5234

4234

1

10

6

4

863

1
396

12

1

1

23

4

2

1

4941219

36
4941219

1

21

1

37

20

15

4

8

4

28

3

22

1049

482

34

6

2

337

1

22

6

1

38

165

1

11

14

1

1

8

48

1

11

1

1

7

10

4

1

5

241

1

1

1

32

1

1

1

1

12

11122

10122

2122

8

1

1

516

448
516

5

63

14331

14331

4

1

1

33

136

36

1

1

143

143

2

1

33

8

1

1

1

10

10

1

1

1

1011412

1214

1214

1214

121

121

121

16

16

16

191

191

191

72

72

72

2301332275235361123

5

5

8541

3

3501

24

1

5

5

441

3

38

31

21

6453

26

35

14

33

10912315101374043
112

522

2

745

1

211

6310116752

1

21

91

1702282

1

2

20

6

18

6

841

13

1

1011466171841

311

11
464617159442

311

21

31472

46

1881

236

16

1111

64112565

2032728

133194

14

1

11

610

6

5

1

1

1532634298983

306195308

53

10115245262

1

49

2

10

1

626
13

613

1

1

217

217

217

524805437472180

48961843746914

1253

52

121

1

111

39716295471

21916

21916

23

23

170

170

39616681

6

391167

511

419852455

3

2

1

3

3

8880

5880

1

2

4

4

9511
1

1

8

1

49

1

1

1

2
332148

1

1

32

5

2

2

6

1123

7

1

1

1

161295

31

22

525

3

64

75

7

7

5

5

1072

1072

6

1012

111

111

2

2

2

1

1

1

2

2

2

4340

4340

2

2340

49

461274104

2154162

10

1154162

2573342

53

272

18142

549

59

59

40

40

2318727

193

7

1

1

5

72

3

3

21

21

18718

2

44

44

332

1

23

1

1

2

1

1

2

2

2

32

9

177

1087112

33710

6

16710

4

4

4

7

19

12

486

486

486

486

3

2816

9

1

72340

72340

72340

41240

3226

38014

311

225

8

78

214

219

262

58

58

58

58

58

333013062112480

9

7
9

2

2591257
728

80257

131

1582

11

59

31231
393558256290

2

3

737

281

99

99

14

3

1

1

36

36

36

19

3

2204

624

1

2

29

14

1

2247

2

19

354

2

6434656190
11

4

1

1134656190

8

1

1

9

18

263145

48

2

128145

5

29

47

4

1

3

6

35
23

12

12

11
12

1

416

17

3

3

3

3

10

2

2

36
4

1

6

6

6

4

2

25

309

5

5

5

5

4

1

2191

29

27

27

27

2

1901

1901

31

1

27

27

10

13

30

361

31

1

10

61

3443515

15

5

5

5

5

1187

1187

1187

1187

3

27099

27099

27099

30

26

30

35

644

1011

4

5682

152

373229

373229

373229
12

32

1

23158

769

3

13

4250338

50

50

200308

2

2

1

130

130

157

9

144

46

46

46

46

1

7

35

1

2

480443141239

11327

11327

152

152

9425

9124

31

4

4

103150

264443121082

1255

103782

16149

16149

16149

42

4011

173798

15723

102

102

4

62

3

7724012

291

291

24

623512

1

1

12

3235

1

15

25

10

15

3722

2

2

2

2

4

9520

9520

9520

9519
9520

1

1

8610

1

59632373605102469767

21

21

1

1

1

1

1

1

1

11

11

11

1

1

1
