# Supplementary material for: Revealing the Microbiome of Four Different Thermal Springs in Turkey with Environmental DNA Metabarcoding
Source: Biology (Basel). 2022 Jun 30;11(7):998. doi: 10.3390/biology11070998 (PMC9311576; doi:10.3390/biology11070998)
Supplement: Supplementary file 1 [file biology-11-00998-s001.zip › Supplementary Data S3/515-806_Merged c2l100 krona/515-c2-l100---ssu---krona----Total---sim_93---tax_silva---td_20.html]

Javascript must be enabled to view this page.

magnitude
magnitudeUnassigned

515-c2-l100---ssu---krona---515d.c2.l100----Total---sim\_93---tax\_silva---td\_20
515-c2-l100---ssu---krona---515k.c2.l100----Total---sim\_93---tax\_silva---td\_20
515-c2-l100---ssu---krona---515n.c2.l100----Total---sim\_93---tax\_silva---td\_20
515-c2-l100---ssu---krona---515ng.c2.l100----Total---sim\_93---tax\_silva---td\_20
515-c2-l100---ssu---krona---515y.c2.l100----Total---sim\_93---tax\_silva---td\_20

86168633757099516258

31175424225

85477969756699481841

71352

71352

7129

7129

7129

62

62

62

790489810239767245

163130133317012

126874824943

1

1

1

1

42

42

257292853

3

2552173

9100

2012

701100

1

1

7084

15

5

5

3382103

338407

1689

7

4

4

4

3382

2382

1

1

56

6

6

4

1

1

196385582

196385582

17283031

11

7910254

1

43

43

2719631

2719631

3811

9

23362

37

37

63

63

3

2

1

3

29

29

29

12

12

12

1

1

1

77417688906597233

17

17

17

708

708

708

7

7

7

4

4

4

4681

4681

42

18

171

237

1

26

26

25

1

5

5

5

14

14

4

1

99858306520

24131563

24131563

46

46

3448274957

144

4

28274953

37

21

16

10

10

1

1

39

39
4

1

1

33

865136030109

51

51

3

3

3

1

1

915

915

13

13

66614749

18

661431

6113

2

18

112

112

14922

53

4

14019

1915055
1

44

1

1715011

5

1

100

55

20

35

45

45

1410157

11015

502

481

1

32

137

7

13

1

1

1

27851
1

16

1

15

1

1

4

4

277

77

2

81

7

1

1

1

1

2

2

24

24

24

781

781

781

24

1

23

541

51

31

1

5100

5100

9

19

13185155115

1

1

1

1

2

2

2

2

2667

2667

120561551

2361

361

1

7

28

1

2

1

1

12

58

3

1

18

18

3

3

4

25

25

4

4

2816

261

2

11

21

2

6
26

1

1

17113

1

1

13

17

355

55

55

3

3

147

133

1

1

1

1

1

31

14

10

10

10

8

2

13637286

6

1253726

112

17

1

7

5

5

816

4

4

112

12

1

7

7

9

9

10

10

10

37

7

3

3

221

10

10

1

1

12

12

112

11

2

32253

11

1

1

1

1

12252

126

2

1

1

1

42

201

85566

564

17

17

17

547

2

85

85

85

85

8

8

8

1004665

4

4

1

1

3

3

84

84

33

32

1

10

4

6

35

6

1

5

17

17

17

17

23

23

23

23

41

21

1

1

2

2

2

1

1

1

1

1

24

6

1

1

1

5

4

2

1

1

1

1

46

46

46

2

1

43

5

331

1

1

1

1

22

22

22

1

2

1

128

128

128

114

14

26

2

13219

1014

94

94

1

34

5

11

11

1

1

3205

1205

2

2

2

70291

220

20

20

2

689

269

28

15

8

7

13

13

14

14

14

1

17876243

17876243

178724

178724

178724

6219

6219

1

6218

20

1

1

1

1

1

155827

8

191

191

191

191

32

32
1

2

2

2

4915

1

1

1

494

494

4

49

1

2

1

3

3

3

3

3

7

7

7

7

7

42

47

2

45

45

45

45

135164867

227641

1

1

27641

3181

1

118

1

1

378225

1

44

22

22

12

1

1

1

24

33
12741

541

86

3

2

2

2

2

2

965717738134

4541863143

11

11

11

25

25

25

48

48

48

932

932

932

1

2939

21

21

2729

2729

8

8

8

2346353

2346322

115

66

1434821

241

31

31

1

1

1

29

5

5

5

5

33

33

32

32

1

27

8

2

2

2

2

4

4

19

19

19

28

28

28

28

1

1

1

1

1

1

1

4915314247

1

1

1

7

5

5

1
2

1

6

6

6

1

1

41531318

41531318

31531316

12

2

366

3

3

23

2

3

5

5

29

29

40

4

1428

1428

313

313

313

313

12

1

3

24

64

70213

70213

70213

7

32

32

3345

3345

24523
10

23803

7

46

9

1180

9

227

944

4

1

1

1
1634

630

630

630

630

4

4

23

1

127

1

5748103

2

2

1

1

61

5

5

5

11

121

121

21

21

1

1

2893

2893
3

2593

2593

20279

8

2

2

2

271

271
1

1

26

18

18

18

11490

2

27

27

11461

20

20

20

1

944

944

944

12

2

42

42

42

2

2

2

2

384891192

159

159

159

1

1

1

22

22

22
20

1

1

1

7

7

7

7

7

7489133

64

64

64

64

5425133

41832

41832

3

41532

6

6

6

5111

5111

5

111

2

1

1

1
