# Supplementary material for: Revealing the Microbiome of Four Different Thermal Springs in Turkey with Environmental DNA Metabarcoding
Source: Biology (Basel). 2022 Jun 30;11(7):998. doi: 10.3390/biology11070998 (PMC9311576; doi:10.3390/biology11070998)
Supplement: Supplementary file 1 [file biology-11-00998-s001.zip › Supplementary Data S3/515-806_Merged c2l100clean krona/515-c2-l100-clean---ssu---krona----Total---sim_93---tax_silva---td_20.html]

Javascript must be enabled to view this page.

magnitude
magnitudeUnassigned

515-c2-l100-clean---ssu---krona---515d.c2.l100.clean----Total---sim\_93---tax\_silva---td\_20
515-c2-l100-clean---ssu---krona---515k.c2.l100.clean----Total---sim\_93---tax\_silva---td\_20
515-c2-l100-clean---ssu---krona---515n.c2.l100.clean----Total---sim\_93---tax\_silva---td\_20
515-c2-l100-clean---ssu---krona---515ng.c2.l100.clean----Total---sim\_93---tax\_silva---td\_20
515-c2-l100-clean---ssu---krona---515y.c2.l100.clean----Total---sim\_93---tax\_silva---td\_20

57514405156726372930

2

2

2

2

2

1

1

1

1

1

1

137175421315

324371130

97

97

97

21

21

21

1

1

1

6

6

6
2

4

1

1

1

1

3437133

373133

36632

36632

3

36332

6

6

6

111

111

111

3

64

64

64

64

55803793156326341485

32

52

45

45

1

3

1

6

2

5

2

12

13

11

2

7

6

6

6

1

20

20

20
9

2

5

4

1

122291

31

2720

20

20

16

11

929

1

1

1

2

2

2

299

2

2

2

2

2

2

2

2

2

54

4

1

3

1

1

5

5

30

1

26

3

2

2

12

12

26

475827

142

2
142

32

8

8

1

1

8

4915

1

1

1

494

494

49

4

1

591

1

1

491

291

2

91

2

2

3

3

3

9

24

52

13

13

13

13

13

40

7831

5

2

2

1

1

1

1

1

1

1

1

4

728

728

728
1

314

2

114

3

572

572

572

2

4

14

1

1

1

23

3

72

1

21428

1

11428

16

37685155115

327561551

21113

11
5

6

1

1

1

2

113

1

4

1

1555

1155

3

1

7

55

2

1

1

2

1

1

15

9216
2

7

3

4

6
106

1

7

2

641

1

1

1

1

21

3

2

1

4

2

1

7

1

151

1

2

1

1

1

3

4

1

3

27361

17

11

1

1

4

6

4361

28

2

7

1

2

1

1

155

10

4

6

1

1

3

25

25

8

5

3

25

18

9

9

5

1
24

7

11

1

4

3

2

1

11

1

1

2

1

1

3

5

5

1

1

1

9

6

1

1

1

1

42

4

4

4

2

2

2

21

21

2

1

1

24

24

24

4

20

82667

82667

1147

114

1033

11

11

1

2

31

6

6

1

5100

5100

842

592253

121

382252

9

242

3

2

2

1

11

11

11

72

826

7

201

1

9

9

8

1

7

1

323

3494665

431

11

201

1

1

2

8

6

2

1

1

8

8

1

1

8
7

1

4

2
4

1

1

152

152

11

46

5

13

2

5

21

58

3

18

37

37

24

13

7

4

4

4

2

2

2

2

1

1

1

47

47

7

7

1

1

39

33

1

2

3

124

89

5

5

5

84

17

1

4

5

1

5

1

7

5

1

1

3

2

1

1

4

1
52

6

1

9

11

14

1

1

6

2

46

46

46

1

2

43

223

223

223

223

217

217

217

217

15

4485214

27212

117

117

117

26195

6

1085

1085

1085

1085

12

15

1

1

1

1

1

1

1

176219

11414

914

914

64

24

60

1

231

231

13

101

62205

9

9

9

14

14
6

6

1

1

37205

1

1

2725717738134

1

1

27

19

19

19

8

2

2

2

2

4

4

33

33

32

32

1

5

5

5

5

1

1

1

1

3

3

3

2

1

28

28

28

28

3

3

3

29

8041863143

18939

9729

8729

1

2

2

1

1

1

1

421

221

1

1

1

1

5
1

4
1

3

32346353

32346322

1115

241

1

166

1434821

31

31

4

932

932

932

14

1

1

13

13

12

3

3

9

4

2

3

1

1

1

11

11

11

148

148

148

1

1

1

1

11

11

5

6

18515314247

946

1

1

3

3

2

4

1

39

39

2
14

1

1

2

3

5

83

3

1

4

3

3

193

2

2

23

9

1

2

1

1

1

26

4

4

22

1

1

1

2

2

3

2

5

2

1

1

1

1

1

1

3

3

3

1

6

6

6

3

161

6

1

2

2

1

1

4

1

1

1

1

51

1

1

2

11

411531318

411531318

2

102

1

4

2

2

1

161531316

3

1

7
8

1

1

1

1
5

1

1

1

1

3

420

4544899

33189
1

36
33089

18

27689

27689

44279

1

271

1
271

1

26

6

6

6

8

12

12

12

25

25

1

1

1

4

6

12

261

16

16

1

14

1

101

1421

6

821

3
1

2

1

1

421

321

1

39

6

4

2

1

1

29
8

4

5

8

4

4

719

230846

230846

23024

23024

1

22924

822

822

1

821

3

9

9

9

1
9

2

4

2

7711490

1

1227

4

1

27

1

1

1

1

1

1

1

12

1
4811461

2

7

7

4

320

320

320

131
61

7

12

1

1944

1944

1944

1842

1842

1142

5

2

13

13

13

10

3

1

1

1

8

8

8

5

311

311

311

16

8

4

4

151

6

2

71

1

642164867

327641

127641

2

3

13

13

3

34
17478225

382

81

81

81

10

624

1

322

1

8

1

2

4

2

1

14

14

44

12

9

2

4

8

1

2

1

124
34541

8

103

24

37

4941

101181

8

2218

20

131

6

5

27

171352

171352

7129

7129

7129

162

1

1

62

62

14353

14353

14353

9

4

5

8
2823

6

38

1183

101

11

17

541

473

50

18

508

391

117

57

57

1

20

20

13634

1

1630

1630

1630

1

630

1

1

1

1

1

1

1

3

1

44

44

1

1

413

413

1

313

313

312

1

2

5

5

5

5

1

3

1

25

25

25

25
14

8

3

1

1

1

1

1

110122794172453245
1

8

8

8

8

3

3

3

3

2481314133148312

446

6

6

7

2

1

3

1

21

4

4

12

7

5

8

1

1

7

3

4

696383322

696383322

2

1

1

17910254

3

1728771

4

4

4

43

43

229

29

29

2

45382

8

7

1

1

2

1

1

2

14

1

1

17382

29

14

14

15

3

3

3

4

339904810333

242

1

42

1

3338663

7

3518

338138

1

1

1

1

15

15

1587592643

2

2

1

7979

3

851173

2412

3

3701100

17084

1

1

1

115

2

2

2
6

4

5

5

2

3

1

1

1

37

37

101529631

2

1

1

21529631

1

19

11462

3811

6

1

3

1

1

12

12

12

1

1

1

2

2

2

493

493
2

1

2

3

1

2

1

1

1

3

4

1

2

1

1

1

1

3

1

3

3

12

2

3

3

3

4

4

4

841965284970233

13

13

5

8

144

3
144

14

1

2

7

1

1

24

24

24

3

3

3

3708

3708

2

1

708

4

32357

1235

1

140

32

63

27

1

1

7

5

5

5

2

2

2

237851
1

5

1

4

1877

77

3

1

1

5

6

1

1

2

2

281

1

11

71

5

5

24

19

5

12

6

6

1

1

6

6

2

2

157

154

2

1

1

1

1

1

5

5

2

3

15

3

3

1

1

11

2

1

4

2

2

1

1
2

1

1

7

7

7

55

11

11

1

8

2

2

2

124

124

1

24

14

14

2

3

9

754906030109

2

915

915

2112

2112

9

3

2

4

613422

1

3

1

263

1

12519

1

3

1

2

2314255

1

314211

3

944

2

1

1

1

2

1

1

25614749

22

18

18

6113

231431

3

413

413

51

51

3

3

1100

55

35

20

45

45

1

1

10185224893

7

1

5

1

2

2

16

16

1444221626

44

1

14

5221622

7

11

11

3

3

3

3

1

1

5

5

12

2

10

1

1

1

1

15

12

3

3413267

3413267

5

5

2

2

1

25

25

5

1

9

10

1

1

2171

2
2171

14

8

53

1

3

137

2

1

3

3

3

3

3

3

217

217

217

57

34

2

10

3

9

2

3

1

4

23

9

4

10

1

1

1

364

273637286

1

1

1

1

1

1

1

1

112

11

2

16

23253726

37

7

3

3

9

9

1

1

1

13221

13

11

2

1

1

12

12

10

10

10

10

10

1

1

1

5816

4

2

2

4

4

112

12

1

1

1

7

7

312

7

7

35

35

1047

5

5

145

145

145

145

22

1

1

2

2

2

2

1

1

1

2

9

9

9

7

2

627
