# Supplementary material for: Revealing the Microbiome of Four Different Thermal Springs in Turkey with Environmental DNA Metabarcoding
Source: Biology (Basel). 2022 Jun 30;11(7):998. doi: 10.3390/biology11070998 (PMC9311576; doi:10.3390/biology11070998)
Supplement: Supplementary file 1 [file biology-11-00998-s001.zip › Supplementary Data S3/515-806_Merged uniq krona/515-uniq---ssu---krona----Total---sim_93---tax_silva---td_20.html]

Javascript must be enabled to view this page.

magnitude
magnitudeUnassigned

515-uniq---ssu---krona---515d.uniq----Total---sim\_93---tax\_silva---td\_20
515-uniq---ssu---krona---515k.uniq----Total---sim\_93---tax\_silva---td\_20
515-uniq---ssu---krona---515n.uniq----Total---sim\_93---tax\_silva---td\_20
515-uniq---ssu---krona---515ng.uniq----Total---sim\_93---tax\_silva---td\_20
515-uniq---ssu---krona---515y.uniq----Total---sim\_93---tax\_silva---td\_20

229074233721215800192301226492

474356906910495130611163643

17866615163911076816145246417
11

2211

147656

1453

2331

2031

2031

15

29

29

32

47

32

271

8

13

30

2

28

28

25

3

24761529925889243486

274

274

274

19

165

11123

1

1

1

1722

79

79

198

52

4

142

512

502

6

4

33

33

400
5

395
19

75

1

300

21124

1

1

2124

2124

1123

1

1

13601803163501297

632421722

14121

41

11

2

11

27

42

1

21

41

4

1

2331

11

2

5

51

72

3

174220

13

1

6

2

4423

34

1

5

7

21

21

21

7

1

1

6

2

4

32

32

22

1

294179412124918

2

2

12

291179312124916

48333

34

27

7

2

36

11

2

84179012021516

32

1

111

6

1

1

33

2

13

18

2155151

13
1275151

111

1

1

4

2

2

2

1

3

33

151

1

1

45

1

1

2

1

2

31

88

4

84

1519

1519

1219

3

236

236

236

6

1

1

1

7272205

2

7

7

1

1102

61

1

4

5

72

8

19

5

51107

3107

1

47

2665

2

5

5

2

1

7

3

165

44844

44844

8

82

42

42

5

5

15

7

2

1047134942409423142

2661

2661

1

35

231

15734211

150342

150316

26

711

711

83

83

4

1

2

2

2

44036

44036

1

4380

226

1401923716715

324

324

13416403

1

6

2

5413403

1

1

131

3

11

941491312312

118

83149131234

3

3

19

19

8

7

1

1

93

1

8

3

1858482388208106

411

411

135822237011678

1

11902181424

32544

41

2

3119445

8310821529045

3

522179128

3

418623

122

21

5122

1

11

11

3

632

27993112

27993112

27993112

1

1

1

7034

11
6734

4

4

19

6

16

7

2

12

2

1

2

1

1

4303

4303

3989

314

10

1807

1807

1807

323

323

1

1

22

1

1

1

1

1

56

6
33

11
27

16

1

1

1

1915

1915

1887

740

1147

28

19

14

5

5

5

1

1

1

1

1599

1126

26

1

1

1

1

1
239

238

362

192

1

12

1

2

2

1

1

15

10

1

4

17

17

16

1

2234

2234

2128

4

4

2

2

1

569

1

1

1

1

31

31

31

31

12

8

8

8

8

248

1768544309110

1768544309110

178591

14

14

38591

38591

685434510

685434510

685434510

963736

963736

717
963736

12

6

3336

4

113

322948321949
2

20893431394

5

4

652331

652331

652331

17

2

2

15

204

1514

43

7

11733555

1
11733555

27

2

1

5

6533514

1

46

21

25

23278

23278

23278

19231
321234

1313
1113

2

1

5

96

9

6

2
735475

2

2382

7442

22

18

1

5

1112

6

111

3

2

41

39

39

39

2

1

21

231

6

6

171

171

1

12

41

6

54

26

582

10

55992288737

793702529

4

361842512

1012

2618425

17

7

218425

63

1869

273

1

2

1

1

223

42

2

1

2955155116

23822

1

1

38

1

37

122

1

22

1412

1412

1

612

7

3

2755182

2755182

581

10551

21

6

4

4

157262

157262
6

10262

113

113

28

13

15

292

1846311

27114

395

10

1

1

1

1

1

2207376933146081573284756

206003531811567669184400

2

89531

9
89531

74

150

3

1

41

51

51

29

22

1

400

400

24

1

352

23

1763112

2331

3

431

16

4

4

14912

97

2

261

121

121

632

3

20
303730751488

11

1

1

421

421

314

311

3

4214

2214

2

134

24

7

2

1

1

6
19013

41

18812

8

2

11612341418
10

5

1753

1

2117

1111

1621

29072

108

1

7

24461296

2

61

3

11

1

1

11

11

2

2

52

52

472

217

1

245

9

7

7

15

15

3211768

1728

21

2

2811

939

6

2

6

72

14

1

57

27

18119

18119

16119

2

11

53241

551

2

7

51

1

4

27

9

4774

455

64

16

1

22929772

1

1

22929771

161

1

592977

122

122

87

10

25

885

885

885

2003127368777732063

184

4

113

31

44

44

29

27

2

16

171121
1

41119

122

1537582535881
21

5

17839

15

442

82

248941301

2

46

764

1

1

1164

2

518

7

241

32884492

4

3

1

2

4

51

2

548

414195

135

81

1997

156

33

11314

13

2

5

71

12

12202

17

70

1025

73111

1

1

69

1

1

1

2

953

1

11

4

1

3

3

16203717458

2180317387

41

7

22347

1

31027

1

1

1025

3

1544132047411694
2

11169129

22348102

199

142312

15206125480

1675

16

1

1

171127

4

1

1

18

11

16913

325451811181286
6

2921155

8

2

262231821

3

1

341

9

1111

2214289108

6

1

2

13

13

3

8

1

1

4760

11

19

4

112219

112219

49

7221

72

72

2

52

34661

34661

34661

1147767

1147767

1

4

28064

69962

38

406

3753

1

14

3

4

16782450106876539336
1

1414
5

5

14

4

9

9

18

18

7

7

614

614

109
1

2

1

2

19

76

1

7

1971

1971

3

89153010537438743

45

11194

32151

235

16

171

2012774

3010533437541

264

264

3

1

2

529201232149328

1

529201232149228

1

1

1

29

29

12

2

5

5

5082721

3012721

206

1

10

5

1

1

2

1

2

23

11

12

12

12

731606118011

5

25
351605710

8322

105

112

14

2

7282

2

1

191

4294

3341801

25

1

631801

1

1

23

1

1

1011129

911129

1

11

33

4

125

11

1

1

4932

31
4932

22

432

397

1911

1911

181

1

1

21

4

4

2

2

191

1

1

181

151

3

11

7

1

3

3

4262038

28

3

5

1

8

3

1

2

5

783

1

782

1461255
6

2277

1967

1

5610

1

1

1

952

146641615304190410356

1

1

1

527

527

23741637

3

501

40

101

22

3

6641634

1

362

54

1

53

1

1

1

45456710011945

33115

513

21

1

21

3

1

2

3

1

2

945669911895

2994

130

24

437491013

28151421

45035

35034

2

5034

1

11

1324

1324

1

113

1010

11

104

104

23

1

8

1231

501

73

73

94

94

1

93

69

69

69

22711854

4253

3253

1

374
1

16

4

14

1

1

4

411

211

1

1

341

14

11

91

3

3

1452

5

5

5

28

28

28

2

2

19

19

19

345

345

3801116011

3801116011
9

25

1

51

19

18131

21

541

12

6

210

51

2

1

5

1

8

3

3

1

5

37

31

1

19

19

1

9

1

32

1

1

1

6

5

1

62

168

6

1

11

1

1

2739

21

1

1

1

31

2

91

52

2

17

25

1224

1224

1

1223

2114

2114

2014

1

986

823641157828099108

4379
823641157828099108

1

2

2

7272689416243

22662

1

365778

227

621

165851205

5821921

2

34121753423

215411602820

14

6416084

1

1

611

5

5

421

1

42

2

1

12

720

612
720

80

22

6

2311

1411

1411

9

31

31

31

381

381

2

1

1

8

3

2413221993859840146

1
1641796507102

65

4

2

1

1

417848342

5

1212422932

42691

2651

34

1

4

4

7904

34

328

14

11

47

285623

95613

131

6

7105835212258

15

83156

22

11908

1

13

1050418335

12

445

1

23

7139

2

5139

11

4

4

1

1

1
9030556107683842

1

1677

61

1121003117

1

22

14

11

1

535

13

4

71

3

32

37

4416308

1

33

71

1930023133815

5

2

8

12

1

1

1

69

44

1

24

3

3

3

4

4

4

4

3

3

3

3

3
33

6

6

6

5

1

24

511

1

1

1

1

1

4187

4187

4187

4186
4187

1

516

516

26

31

31

31

354818431182665

2355

1963
23

39

2

7

233

18

4

80

6

25

15

10

1

9

82

114157214

4115721

1915720

1

1

1715720

1

1

1

3
2

1

171

9

1

7

1

734

25582473

149
25582473

7

24022473

24022473

23266

143

143

143

8966

409168697117

28059

1159

11

59

269

27

8

218

3

2

11

69

416861447

31147
416861447

1

1427

1

258

3

31

31

31

4

901

86

86

1

1

31

31

24

3

3

21

21

1

4

14662148537

133521169

7241943

17

17

143

143

257

257

4381

2551

76

107

281

1

271

188

188

187

1

4022126

5

5
2

2

1

6

6

6

1

1

1

9

9

9

12831611

5014

31
21

1

1

73

230111

230111
230011

1

326

1046

1046

1046

4

4

4

4

450527753311

450527753311

2735331

2735331
48

264

733681

8

45

4505040001

4

4

61
4504640001

3786510

4125334891

1

13266339

13266339

13266339

58

58

281486

233206

4574

254

4

251

73

632554

14

632414

45

17

13

15

601

601

4006929
968

271

3806927

27

481

2332

20

52

52

69

69

38

38

3

3

3

3

3

1424

1424

1424

171
1424

3

2

153

105

1

1

1

1

24
30

6

6

6

11

100548020417

6493913

853353

853353

2933

5635

56456

56456
3

64

358

13956

35647711314

139

139
20

5

114

13747711211

591

591

591

13

20

1

375949

2435648

2435648

14

1021

1

1

1

1

416320381812259

3

1112
3357367

172

72

15

128367

1858

1

6

5
6

1

4

3

8441397328
13

2

22

9

1241397327

20

1

3

1

2

450103584961
3941

24

20

633

2

8437

35

1

69

69

69

101

20

7942

3397451

22

838

12

1

3

22

1

21

1159

1

60

13

255223970

31

545

61

9962

472

10

98223

10154

53336

3754630

5

5

1

1

4

4

12

14

30194

30194

30194

4

823

28

1010

44

7079

1

132

35

14

364349

364349

1
364349

7121

31

11

43

20227

2187

2187

2187

2187

5

184

2

2

2

2

2

48184

48184

315
318

3

45166

45166

129

49

40

128

38644231469

85141

7813

7813

8

8

10

10

685

675

1

22344231315

49

164789

3925

19025

5520131

6

18

11

7

26

26

320131

1

1

20131

1

2

45

45

4

5

8374

8374

8374

3115

171

111080

1

371

276223225311532137

416036

416036

160

160

436

224

224

7

7

217

192

3

22

1

4056218871

4056218871

2403151053113586

25553971

133

7

361

1

1

353

3

53

13

2252
215

102

111

3914754

3

3

7

5

2

101

1

1

9

91474

1

1

1474

7

1

1

73

5

2

2

1

3

3

6

4

1139124

474

43

14

3

33

6

10

4

6

2

2

11

11

32

32

1041
2

83

11

15

3

8

92

92

19

2

5

10

2

35016

35016

72

29

43

1

1

119

402

273

24

249

3

3

14586885

221

1095

11

82

11

55

2
1486780

3

2302

1

1

3

77

14

13

80

471

1

197

61834142

57138
138

10

3

34

10

494343

2

8

19

2

1

31

2

2

85342

14

1

4

5

3

18

87

237

1

18

15

3

9

2

2

1

1

3

261

10

10

4

33901

33

33

33

901

901

90

1

581142

521009

151

151

3

544

3

4323

3

4320

390

21

6133

68

68

68

50
68

15

1

2

13288

105

105

105

81

21

3

3

34119

19116

185207546513928

453

2946

3720754744

3720754744

3720754744

3720754744

11446113085

346206

346206

346206

111112879

1

1211

11

11

11

1

1

21

1

8

109254

109254

109254

63754

59023

4731

455

4

2

89

360

14

2

2

2

2

1

1

618241

2222

6

6

1

210

210

210

210

8

1889

789

3

3

3

2

6

1

1

1

1

2

7442

5356181

5

5

5

114

2232412

18

18

1204393

28

28

1

1

1

411

411

411

411

12

271

2

19
39197221777

75

3

25

5

2

2

31581059

31581059

31581059

13

1

3

1571055

13

1

2391139
3

1

2

5

3

316

710

710

710

392

392

392

767

31

31

1

1

2

351

16

16

16

534

1

41

4

4

4

1

91640

91640

2951069558637215

2

2

2

2

191847324

73

186

75

31

31

8

1

1

6

18417

1

1

1

1

1

2117

26088555856126

11

11

1

1

3

3

3

148

148

1482

1482

6

1422

95

9

9

5

5

2472

72

24

24

2

447211

447211

2

247211

381212435113

2

2

4
241239

2

17

41

1

1

211

1

1

2

1

1

1

1

1

3

3

101299

1

2

51299

2

10811
11

79

9

1

1

132813

513

4

32

33

2

1

502115107

25121455

64

15255

4119

2590152

1890152

7

1

1

1

1669356431

1

1

2509

2509

1649821

160921

6

411

198

198

32

2

30

1

44

433

33

2

2

3

2

1

1

1

13

440

38

38

42

42

46

46

46

46

46

196

5

341157

347851087212935

2571129

51
50

1

1613

1613

512

31

8

143110

3110

3110

5

76

76

59

59

4716

453

453

453

1

9

1

343

54820

887

887

887

46013
2

4

4

60
4

32

8

2

2

2

1

9

17

17

147

1

9

3

4

2

7
3536

14

88

18

2

1

1331

16

53

12

95

1648271

10343

10343

10343

10343

71

610

610

610

1

510

101

101

91
3

1

3

2

1

1

111207

111207

111207

111207

282143

282143

25543

20743

35

11

2

2

2

241

241

1

1

221814

221814

3841

1421

239

3

615

111

504

457

7624
10

8

46

84

604

104

4

4

3

3

1

108724

108724

108724

6716

1054

31024

1

35

223

2

2973130138123816432

52513008634212746

285105104212719

51

21

2

1

14589

39999644

39999644

81

39918644

4

4

31831

261
31831

1177

11

2542674212064

2542674212064
33133

653

115235

412644211993

7

7

7

7

249663

28

28

28

2496

2491

2491

5

5

35

35

35

240220

16181

91

91

1

1

9

9

1

1

6

6

5

5

1

1

18

18

18

1

17

14

14

14

14

163617

163617

113617
163617

5

189855

189855

1540
189855

111

775

755

2

10

114

465

101196

101196

1

1

1

2

28

3

196

196

196

67

44

4

1

3

1166

1166

1166

1166

1166

215

215

215

228

224

224
217

7

4

1916

18

18

18

116

116

14

14

12

2

1

1532

1532

1532
