# Supplementary material for: Revealing the Microbiome of Four Different Thermal Springs in Turkey with Environmental DNA Metabarcoding
Source: Biology (Basel). 2022 Jun 30;11(7):998. doi: 10.3390/biology11070998 (PMC9311576; doi:10.3390/biology11070998)
Supplement: Supplementary file 1 [file biology-11-00998-s001.zip › Supplementary Data S3/515-806_Reverse c2l100 krona/515r-c2-l100---ssu---krona----Total---sim_93---tax_silva---td_20.html]

Javascript must be enabled to view this page.

magnitude
magnitudeUnassigned

515r-c2-l100---ssu---krona---515dr.c2.l100----Total---sim\_93---tax\_silva---td\_20
515r-c2-l100---ssu---krona---515kr.c2.l100----Total---sim\_93---tax\_silva---td\_20
515r-c2-l100---ssu---krona---515ngr.c2.l100----Total---sim\_93---tax\_silva---td\_20
515r-c2-l100---ssu---krona---515nr.c2.l100----Total---sim\_93---tax\_silva---td\_20
515r-c2-l100---ssu---krona---515yr.c2.l100----Total---sim\_93---tax\_silva---td\_20

1651918460165821344815112

2

2

2

2

2

2

2

2

2

1632717350165601343413160

20087569159831390376

1891361269481098347

28

28

28

39

31

2

29

8

1

6

1

19

19

19

110

110

10

1

30

30

1

29

2

2

2

418

418

418

60

5
60

8

2

45

1

21535

21535

1

1

717

44

774

23

23

1

22

1132

1

1

90

26

64

42

42

222816

328

28

3

1916

8

1

1016

3

3

2

1

1

1

1

304166131
1

1

1

6

6

1

1

26

26

1593

93

1

14

188

188

1

1

271101

1510

23

233

1

4

4

813

8

3

1

52

21

30

1

1

1

1

2

112519

112519

1

12519

1391546104150141

8

8

7188362
3

1

35

1

1

4352

5321

3

4

281

24426

1

4

2

4459367

1

1

2559266

61

12

212

212

54954957

23

1934

27

52

9523

1127

121

6

1

217

17

2

36

13

23

14

7171

6771

4

57

57

181

2092126726930

312

312

12

12

1

1

4

4

281634779922

222

1633

54774922

1

1

1

24
105

10

46

25

29

29

13

13

14919186

14919186

13

13

1111

104719

104719

571

1919

84

373

2

2

2

1

2

2

2

4

25

25

25

1173957903529229

5310

5310

10

53

1

1

1

15

15

15

108

4
1

3

1

1

4

1

1

8

8

11318650868814

3

3

38

33

5

6

6

7114132

102

1

8

22

10

611003

6

5

1

33062516207

3

315820

1

305

30323497

124385652

34291

62

13

1293764

241

241

436837201376

436837201376
7

2

2

162484

24

211

26137312

13363046104

46

1

521421

521421

1411

2

25

2

25

8

43

43

43

5

5

5

30

30

30

8

4

4

4

42

42

1

41

11

11

11

291107191

291107191

32

3831

25372

19

26538

5538

8

8

13

13

25

1

4

3582965111

3

3

3

3

255

255

135

79

56

48

13

35

6

66

10

22

34

30

29

29

29

29

32

4

17

4

4

13

6

7

2

9

9

9

7

1

1

1

1

29656

29656

29656

6

2885

80

42

41

34

12

5

1

1

15

1

1

6

5

1

1

1

1

123

123

123

123

1

1

1

1

1821

1821

1

1

1721

4

1321

883

883

883

48

7

41

403

73

33

4832772362262

7163

2138

9

9

2

2

72

57

525

26364

26364

120

20

20

20

1

1

1

31

31

30

3

27

1

1

442161236227

618527

1

5

1

4

18527

13

29

27

143

28

42332

21

12

2

32
40

8

2

2

2631

10

10

4

2

2

19

12

1

6

17

17

7
9

2

2

68

38

30

27

521

521

46

12

30

4

5

3

1

1

12154

154

154

6

2

4

6

6

91717

577

1

1

3

36

12

47

17
1217

8

4

1

1

21

338

338

33

33

8

2434912

7301

52

281

281

2

235191

4316

23

23

23

16831

25

25

1

1

3

3

11

11

132

13

18

101

1

1

1

11

26

120346

120346

120346

15

20

20

24

24

35916
208

397

28446

30

69

43

63

63

4545

50

4495

1

1

9

9

34

3721

3328

359

34

11

1815

9601167

5601137

34

1

5

80

242

1271

1

1

271

271

946

946

946

30

4

4

1

1

3

3

1

36

68728269

68728269

7653

7653

7652

1

6872616

6872616

1518

6857598

6255211

277

246

246

1

20

36

31

31

11

2

3555141

34

34

9

25

155141

134

1

34

34

34

34

7

2351428116149460

95

25

25

25

25

8312745311910

611617

432

432

67315

67315

11

11

1

10

3

25

25

25

1

1

1

219

219

7

212

14142711810

1191

1191

1414161099

36

736781

271

127810317

581

581

1

32

26

1553

1553

1553

633

633

22

611

2

2

25

21

11

1

1

1

1

1

1

1

25

25

2

1

1

4

4

160

84

84
14

26

26

18

76

16

16

7

7

3

3

50

50

148154633038

2811

1911

1

1

1

1

2

1

2

12

9

8

1

32

32

32

2

241183224

241183218

95

41182718

1

9

1

6

165

3

62

1

61

1

1

9230

104

34

1

6

11

413

4

13

4

5513

5513

8

8

136

136

136

1

1

1

1

81

81

15

66

66

17

17

19711912165

129140

4
129140

125140

125140

111

41
101

3

3

1

1

43951021

7

7

7

2

2

2

953

953

66

3

29

6

6

6

308

18

8

1

29

1

1

4

23

18

2242

1242

1

1

242

242

1

123

23

10

10

10

7

3

10

10

10

10

10

23

2

9013580

9013580

1316

1316

1316

90564

90564

90564

27

27

25

25

25

2

6

6

6

3

1

2

1

1

1

91

91

91

91

2

7

1

19

2

2

2

3184

2195229

1095142

244
243

1

651

651

651

30

30

30

770

770

7

463

3

127

1

9

9

9

2

7

10

268
1

66

1

1

1

8

27

27

27

16

39

39

39

39

128

339160476832

11559

1

1

3

1555

79
1082326651

61

283582

18

1

36

2

42990

6

69

15

1

30456
63

223

8

1

956

2422122

2

1120

5

1

1722

38522

38522

121

121

12

11
12

1

1

1

1

423159
4

77

77

12367

12367

12367

1

1

2366

9
1

1

1

1

4

3

1

1

2

11

1

1

1

3978451

2518

24112

1

1151

1151

1151

2

26

37217

12

2

2

1

1

3715

3715

15

37

1

724

724

24

7

7

5

19

19

19

19

26

4337

2832

15

15

5

5

46123617324

272

271

1

1

1

6

6

6

469661731

612

6

6

12

19

19

19

41

41

41

22

22

35191

14

14

213

20

1

3

1

1

16

16

330

114

114

216

16

2

43442

15

15

43

43

227

227

2

2

15

15

15

15

17

17

17

17

248

17398

1

1313

1313

1313

2

3

13

6

2

3351

3351

3351
309

323

19

34

34

34

34

268

2

5

63

63

63

63

1923452896

5234550

5234550

5234550

5234550

218

7

1252321

122279

542

542

542

66105137216

1241005971736

4

4

4

3

3

3

3

3

37

37

7

7

7

1

2

218

218

217
218

1

6

1

5

5

1

1

1

12

12

12

12

12

21958261510

5

1665721510

26

26

26

162521419

162521419

22521419

14

585

2191

2191

15

691

3016

5

5

5

1

1

1

301

301

301

77471

77471

63
77471

2

147

51

41

1

6

21

2

2

1

1

1

1

1

1

1

1

2

2

2

2
