# Supplementary material for: Revealing the Microbiome of Four Different Thermal Springs in Turkey with Environmental DNA Metabarcoding
Source: Biology (Basel). 2022 Jun 30;11(7):998. doi: 10.3390/biology11070998 (PMC9311576; doi:10.3390/biology11070998)
Supplement: Supplementary file 1 [file biology-11-00998-s001.zip › Supplementary Data S3/515-806_Reverse c2l100clean krona/515r-c2-l100-clean---ssu---krona----Total---sim_93---tax_silva---td_20.html]

Javascript must be enabled to view this page.

magnitude
magnitudeUnassigned

515r-c2-l100-clean---ssu---krona---515dr.c2.l100.clean----Total---sim\_93---tax\_silva---td\_20
515r-c2-l100-clean---ssu---krona---515kr.c2.l100.clean----Total---sim\_93---tax\_silva---td\_20
515r-c2-l100-clean---ssu---krona---515ngr.c2.l100.clean----Total---sim\_93---tax\_silva---td\_20
515r-c2-l100-clean---ssu---krona---515nr.c2.l100.clean----Total---sim\_93---tax\_silva---td\_20
515r-c2-l100-clean---ssu---krona---515yr.c2.l100.clean----Total---sim\_93---tax\_silva---td\_20

77365767400329435552

12459297493

1

1

1

4

4

4

77474

77474

77474
633

2

147

51

1

41

6

37

37

1

2

7

7

7

103

103

102
103

1

3

3

3

3

3

6

1

5

5

12

12

12

12

12

21

1

1

1

1

2

2

1

1

1

1

2

2

2

2

2154526379

1356

5

5

5

135

135

135

1

1

1

164102379

341

1891

1891

391

15

16252288

16252288
14

2252288

26

26

26

5

61103137141

75495072398129294918

19

7413196

7413196

74180

74180

74180

1316

1316

1316

27

27

27

39

39

39

39

9601148

5601118

61

1

242

946

946

946

5

1271

1

1

271

271

34

30

4

4

1

1

3

3

2139229

1039142

770

770

3

7

463

91

91

91

127

244
243

1

30

30

30

9

9

9

7

2

10

1

268
1

1

66

1

1

8

4

10

10

10

10

10

19710912110

2242

1242

1

1

242

242

1

111

1

1

101
41

3

3

43851021

18

2

2

2

6

6

6

308

29

1

1

4

23

18

1

8

853

853

29

56

3

7

7

7

123

23

10

10

10

12985

4
12985

12585

12585

129123533432586358

117411051074333329

25

25

25

1

1

1

28

28

28

2

2

2

60

60
5

45

8

2

232816

328

28

3

2016

8

1116

1

2

2

2

1111

1
178128131

813

3

1

8

172101

33

25

1

11

1310

62

62

2

2

1

1

6

6

26

26

1555

14

55

1

52

1

30

21

4

4

39

8

6

1

1

31

29

2

181

1

418

418

418

4

1

46519

46519

286

1819

96

65

2

2

2

207131835165

312

312

12

12

29

29

103
24

44

25

10

4

4

13

13

13

13

1

1

1492826

1492826

1

1

2882524157

222

1

5521157

821

1

1

1

19819

19819

9819

1

3

3

2

1

30

30

29

1

19

19

19

1120

42

42

78

26

52

1

1

2239

2239

1

108

1

87

44

2

110

110

10

1

131481121150135

8

8

1212

1

212

46704957

7023

23

1934

27

44

40241

3971

117

34

11

23

3
2108362

30

1

2

1

5321

802

1

35

6926

1

1

2

261

2

1127

1

6

121

459461

99

8

279352

1

1

55

55

14

217

17

2

23

23

1

22

1171248235825329

11

11

11

15

15

15

108

1
4

3

1

1

1

1

8

8

4

5

5

5

30

30

30

111105191

111105191

7370

3831

32

19

41978111066

41978111066
7

2678312

16894

2

3

2

116560573

27

2

5310

5310

10

53

26538

8

8

13

13

5538

52821

52821

1

30811

2

19

8

2

1

1

1

1183413208014

6

6

37

32

5

7112432

1

8

71833

22

102

6

5

1

3

3

241

241

3710355207

6842837

36520

263

1

3

12798572

1218656

13

62

5931

42

42

1

41

8

4

4

4

43

43

43

4
223140

1

1

9
1

4

1

1

1

3

11

1

1

1

12348

12348

12348

1

1

2347

77

77

248

2064912

7301

281

281

2

52

198191

23

23

23

13131

1

1

11

11

95

13

18

64

3

3

25

25

1

1

1

4316

11

26

16

17156

1313

1313

1313

6

2

2

3

13

34

34

34

34

1

3109

3109

3109
67

323

19

358491111

1

1

1

1

4916

4916

4916

6

436

55

30

1

1

1

1

1821

1821

1

1

1721

1321

4

123

123

123

123

28

5

5

5

2

4

17

4

4

13

13

42

41

1

1

6

1

4

1

34

17

10

1

1

5

1

1

1

7

259

259

70

23

9

38

6

135

79

56

48

35

13

29

29

29

29

3

3

3

3

19

19

19

19

128

268

2

5

63

63

63

63

27

27

25

25

25

2

6255211

3555141

155141

34

34

9

25

277

246

246

36

1

20

31

31

2

11

1

56123617324

15

6

6

6

10272

271

101

1

1

10

10

469661731

35191

1

1

16

16

14

14

213

20

1

3

41

41

41

19

19

19

330

216

2

16

114

114

22

22

612

6

6

12

43442

227

227

15

15

2

2

43

43

1

26

6

6

6

1

3

2

1

1

6661426

6661426

1312

1312

1311

1

666114

666114

66495

219

2

2

2

235709116149457

148148633038

9330

11

4

5613

5613

8

8

413

4

13

104

7

34

241123224

241123218

1

1

61122718

75

9

6

32

32

32

165

3

62

1

61

1

1

2

136

136

136

2711

1811

1

2

2

1

11

1

1

1

9

1

8

25

835615311910

11

11

1

10

611617

67315

67315

432

432

1553

1553

1553

581

581

32

1

26

1

1

1

219

219

212

7

112

112

90

22

3

12222711810

1222161099

716781

271

112110317

3

1191

1191

25

25

25

1

1

1

1

25

25

4

4

2

1

1

2

2

78

78

15

63

63

17

17

95

160

76

7

7

50

50

16

16

3

3

84

84
14

26

18

26

25

25

25

25

21

1

1

1

11

1

1

1

1

7

1

883

883

883

48

41

7

403

73

33

17

17

17

17

23

7

15

15

15

3978451

37217

12

2

2

1

1

3715

3715

15

37

1

724

724

7

7

24

5

2518

24112

26

2

1

1151

1151

1151

2

4781932082254

338

338

33

33

8

26364

26364

120

1

1

1

20

20

20

31

31

30

27

3

1

1

2155

25

2130

51

2

2

9

9

70

44277208227

1270

6

4

2

70

70

6

1

5

2631

3

46

12

30

4

1

1

2

68

38

30

27

4

2

2

17

17

7
9

2

19

6

12

1

10

10

521

521

5

42332

40
32

8

21

2

2

2

12

91717

577

3

47

36

12

1

1

1217
17

4

8

21

1

1

615727

15727

115

29

13

27

1

5

4

1

28

4337

5

5

15

15

2832

38179

38179

3

36

25

134

34

34

34

34

1

182635848

125690

541

541

541

12649

7

5263133

5263133

5263133

5263133

118

3177

339160471352

242256

1722

2

154

1

5

11559

3

1

1555

1

63
30456

956

8

1

223

1082321237
79

2

36

69

4600

28558

1

15

18

61

6

1

121

121

12

12
11

1

1

1

1

91

91

91

91

2

7

1

40066

40066

40066

15

20

20

205
12196

7026

70

211

31

1185

1136

49

9

9

1

1

11

24

24

34

1426

1256

136

34

62

62

2015

2

2

2

2

2

2

2

2

2
