# Supplementary material for: Revealing the Microbiome of Four Different Thermal Springs in Turkey with Environmental DNA Metabarcoding
Source: Biology (Basel). 2022 Jun 30;11(7):998. doi: 10.3390/biology11070998 (PMC9311576; doi:10.3390/biology11070998)
Supplement: Supplementary file 1 [file biology-11-00998-s001.zip › Supplementary Table S1.pdf]

Supplementary Tables S1. Results of physicochemical analysis

a: Physicochemical analysis of Nevşehir-Kozaklı thermal spring

**Parameter**	**Measurement Value**
Temperature	89.1°C
pH	7.5
Bicarbonate	555.1 mg/L
Chloride	730 mg/L
Fluoride	3.35 mg/L
Sulphate	587 mg/L
Nitrate	13.7 mg/L
Silicate Acid	72.1 mg/L
Arsenic	0.226 mg/L

b: Physicochemical analysis of Ankara-Kızılcahamam thermal spring

**Parameter**	**Measurement Value**
Temperature	86°C
pH	7.2
Bicarbonate	1586 mg/L
Chloride	229.14 mg/L
Fluoride	1.57 mg/L
Sulphate	100.78 mg/L
Nitrate	8.44 mg/L
Silicate Acid	69 mg/L
Boric Acid	38.22 mg/L

c: Physicochemical analysis of Yozgat-Boğazlıyan thermal spring

**Parameter**	**Measurement Value**
Temperature	52°C
pH	6.25
Bicarbonate	852 mg/L
Chloride	1180 mg/L
Fluoride	1.98 mg/L
Silicate Acid	51.3 mg/L
Sodium	667 mg/L
Calcium	276 mg/L

d: Physicochemical analysis of Muğla-Dalaman thermal spring

**Parameter**	**Measurement Value**
Temperature	26.9°C
pH	6.54
Bicarbonate	857 mg/L
Sulphate	1014 mg/L
Chloride	8463 mg/L
Silicate Acid	16 mg/L
Sodium	4092 mg/L
Arsenic	0.22 mg/L
