# Supplementary material for: Revealing the Microbiome of Four Different Thermal Springs in Turkey with Environmental DNA Metabarcoding
Source: Biology (Basel). 2022 Jun 30;11(7):998. doi: 10.3390/biology11070998 (PMC9311576; doi:10.3390/biology11070998)
Supplement: Supplementary file 1 [file biology-11-00998-s001.zip › Supplementary Table S2.pdf]

Supplementary Tables S2. Results of Qubit measurements

Assay Name	Test Name	Original sample conc.	Units	Sample Volume (μL)
dsDNA High sensitivity	Negative Sample 16sV3	11,4	ng/μL	2
dsDNA High sensitivity	Negative Sample 515F-806R	15,1	ng/μL	2
dsDNA High sensitivity	Nevşehir 16sV3	64,8	ng/μL	2
dsDNA High sensitivity	Nevşehir 515F-806R	55,3	ng/μL	2
dsDNA High sensitivity	Ankara 16sV3	14,5	ng/μL	2
dsDNA High sensitivity	Ankara 515F-806R	10,9	ng/μL	2
dsDNA High sensitivity	Yozgat 16sV3	22,3	ng/μL	2
dsDNA High sensitivity	Yozgat 515F-806R	42,1	ng/μL	2
dsDNA High sensitivity	Muğla 16sV3	13,9	ng/μL	2
dsDNA High sensitivity	Muğla 515F-806R	18,6	ng/μL	2
